# Adiponectin Gene Polymorphisms: A Case–Control Study on Their Role in Late-Onset Alzheimer’s Disease Risk

**DOI:** 10.3390/life14030346

**Published:** 2024-03-07

**Authors:** Juraj Javor, Vladimíra Ďurmanová, Kristína Klučková, Zuzana Párnická, Dominika Radošinská, Stanislav Šutovský, Barbora Vašečková, Veronika Režnáková, Mária Králová, Karin Gmitterová, Štefan Zorad, Ivana Shawkatová

**Affiliations:** 1 Institute of Immunology, Faculty of Medicine, Comenius University in Bratislava, 811 08 Bratislava, Slovakia; vladimira.durmanova@fmed.uniba.sk (V.Ď.); zuzana.parnicka@fmed.uniba.sk (Z.P.); ivana.shawkatova@fmed.uniba.sk (I.S.); 2 Clinic for Children and Adolescents, Faculty Hospital Nitra, 950 01 Nitra, Slovakia; kristina.kluckova@fnnitra.sk; 3 Institute of Medical Biology, Genetics and Clinical Genetics, Faculty of Medicine, Comenius University in Bratislava, 811 08 Bratislava, Slovakia; dominika.radosinska@fmed.uniba.sk; 4 1st Department of Neurology, Faculty of Medicine, Comenius University in Bratislava and University Hospital Bratislava, 813 69 Bratislava, Slovakia; stanislav.sutovsky@fmed.uniba.sk; 5 Department of Psychiatry, Faculty of Medicine, Slovak Medical University in Bratislava and University Hospital Bratislava, 826 06 Bratislava, Slovakia; barbora.vaseckova@szu.sk; 6 Care Center Centrum MEMORY, 851 03 Bratislava, Slovakia; reznakova@centrummemory.sk; 7 Department of Psychiatry, Faculty of Medicine, Comenius University in Bratislava and University Hospital Bratislava, 813 69 Bratislava, Slovakia; maria.kralova@sm.unb.sk; 8 2nd Department of Neurology, Faculty of Medicine, Comenius University in Bratislava and University Hospital Bratislava, 833 05, Bratislava, Slovakia; karin.gmitterova@fmed.uniba.sk; 9 Institute of Experimental Endocrinology, Biomedical Research Center, Slovak Academy of Sciences, 833 06 Bratislava, Slovakia; stefan.zorad@savba.sk

**Keywords:** adiponectin, Alzheimer’s disease, association, *ADIPOQ*, single nucleotide polymorphism, susceptibility

## Abstract

Adiponectin, a hormone secreted by adipose tissue, plays a complex role in regulating metabolic homeostasis and has also garnered attention for its potential involvement in the pathogenesis of late-onset Alzheimer’s disease (LOAD). The objective of this study was to investigate the association of *ADIPOQ* variants with plasma adiponectin levels and LOAD risk in subjects from the Slovak Caucasian population. For this purpose, 385 LOAD patients and 533 controls without cognitive impairment were recruited and genotyped for a total of eighteen *ADIPOQ* single nucleotide polymorphisms (SNPs). Both single-locus and haplotype-based logistic regression analyses were employed to assess the association of SNPs with LOAD risk, while linear regression analysis was used to explore their influence on adiponectin levels in LOAD patients. *ADIPOQ* variants rs822395 and rs2036373 in intron 1 were found to significantly elevate total adiponectin levels after accounting for several potential confounders. Additional SNPs in the 5′ region and intron 1 exhibited a non-significant trend of association with adiponectin. However, none of the *ADIPOQ* SNPs showed an association with LOAD risk, neither in the whole-group analysis nor in subgroup analyses after stratification for sex or the *APOE* ε4 allele, a well-established LOAD risk factor. In summary, while adiponectin has emerged as a potential contributor to the development of LOAD, this study did not unveil any significant involvement of its gene variants in susceptibility to the disease.

## 1. Introduction

Late-onset Alzheimer’s disease (LOAD) is a multifaceted neurodegenerative disorder and the leading cause of dementia in people over 65, accounting for an estimated 60–80% of cases [1]. The disease is characterized by excessive accumulation of abnormal amyloid beta (Aβ) and hyperphosphorylated tau (p-tau) proteins in the brain, leading to the formation of extracellular senile plaques and intracellular neurofibrillary tangles, respectively. The presence of these protein aggregates, combined with additional pathological hallmarks like oxidative stress, mitochondrial dysfunction, cerebral glucose metabolism deterioration, and neuroinflammation, ultimately results in neurodegeneration and cognitive decline [1,2,3]. The pathogenesis of LOAD is complex and involves an intricate interplay of genetic, environmental, demographic, and modifiable lifestyle risk factors [4]. LOAD has a substantial heritability of approximately 70% and features a genetic architecture defined by multiple interacting genes [5]. Recent meta-analyses of genome-wide association studies (GWAS) have significantly expanded the number of known disease susceptibility loci, totaling up to 90 [6,7,8].

Midlife adiposity and late-life weight loss are frequently linked to a higher risk of cognitive decline and AD in observational studies [9,10]. However, the exact nature of this relationship remains a source of controversy and may even involve reverse causation between a decline in body mass index (BMI) in later life and AD [11,12,13,14,15,16,17]. Beyond serving as a depot for energy storage, white adipose tissue also functions as an endocrine organ, secreting a variety of biologically active substances. These molecules are collectively known as adipokines and play a pivotal role in regulating whole-body metabolism and inflammatory responses [18]. Adiponectin, a 244-amino-acid-long protein hormone, stands out as the most abundant adipokine found in plasma. It regulates glucose and fatty acid metabolism and exerts anti-diabetic, anti-atherogenic, and anti-inflammatory effects [3,19,20]. Despite being produced mostly by adipocytes, circulating adiponectin is inversely correlated with BMI, and its levels are decreased in conditions defined as metabolic risk factors for AD, such as insulin resistance, type 2 diabetes mellitus (T2DM), obesity, hypertension, and cardiovascular diseases [21,22]. Adiponectin signaling has shown various neuroprotective effects in experimental settings, including improved cerebral insulin sensitivity and glucose uptake, promotion of neurogenesis, reduction in oxidative stress and inflammation, prevention of blood–brain barrier (BBB) disruption, inhibition of Aβ accumulation and hyperphosphorylation of tau, and regulation of synaptic plasticity and cognitive function [3,18,21,22,23,24,25]. On the other hand, studies on circulating adiponectin in human subjects have not yielded unanimous results, with the majority of them, including a recent meta-analysis, reporting significantly higher blood adiponectin levels in AD patients compared to participants without cognitive impairment [26,27]. Moreover, recent evidence has indicated that elevated adiponectin might be associated with the severity of Aβ accumulation [28,29], highlighting the potentially detrimental involvement of adiponectin in Aβ amyloidogenesis and neurodegeneration in aging [22,30,31].

Circulating adiponectin has been shown to exhibit substantial heritability ranging from 30% to 70% [32,33,34,35,36,37]. Genome and exome-wide association scans have provided evidence for more than 30 loci robustly associated with plasma adiponectin levels [35,36,38,39,40,41,42,43,44,45,46,47,48,49,50,51]. The top prioritized gene in Caucasians is the adiponectin-encoding gene *ADIPOQ*, located on chromosome 3q27.3 and spanning 15.8 kb. *ADIPOQ* is composed of three exons and harbors numerous single nucleotide polymorphisms (SNPs) that have been extensively studied in different populations [52,53], with several of these variants showing associations with alterations in adiponectin levels and metabolic syndrome-related phenotypes [33,35,36,37,38,39,40,41,42,43,44,45,46,47,48,49,51,54,55,56,57,58,59,60,61,62,63,64]. The intricate effects of adiponectin in the nervous system have raised the hypothesis that functional variants in *ADIPOQ* could be involved in susceptibility to LOAD. Two studies exploring this assumption have indeed reported an association of *ADIPOQ* polymorphisms, namely, −11,377 C>G (rs266729) in the gene promoter and +276 G>T (rs1501299) in intron 2, with LOAD risk in the Chinese population [65,66]. However, there is currently no evidence supporting the involvement of *ADIPOQ* variants in the predisposition to LOAD in Caucasian populations.

This study aimed to further elaborate on the role of *ADIPOQ* in genetic susceptibility to LOAD by assessing the association of eighteen common SNPs across the *ADIPOQ* gene with LOAD risk in the Slovak Caucasian population. Additionally, our aim was to explore the potential impact of sex and the major LOAD risk factor, the apolipoprotein E gene *(APOE)* ε4 allele, on this association. Finally, among individuals with the disease, we analyzed the influence of *ADIPOQ* variants on the age of AD onset and adiponectin levels.

## 2. Materials and Methods

### 2.1. Study Participants

A total of 918 Slovak Caucasian individuals from the western regions of the country were recruited for the purposes of a study investigating risk factors for LOAD. The patient group comprised 385 unrelated subjects (243 females and 142 males) diagnosed with the late-onset form of the disease at the neurology and psychiatry departments of the University Hospital Bratislava. The diagnosis of LOAD followed the National Institute of Neurological and Communicative Disorders and Stroke and the Alzheimer’s Disease and Related Disorders Association (NINCDS-ADRDA) criteria [67]. The ethnically and geographically matched control group consisted of 533 unrelated volunteers aged ≥ 65 years (320 females and 213 males) without cognitive impairment and with no family history of AD or other types of dementia among their first-degree relatives. Trained investigators assessed multiple cognitive domains in study participants using the Montreal Cognitive Assessment (MoCA) screening test [68], where scores ranged from 0 to 30, with 26 or above considered within the typical range. In addition, information on selected demographic and clinical characteristics was collected, including body mass index (BMI), type 2 diabetes mellitus (T2DM), and hypertension (Table 1).

Written informed consent for study participation and personal data management was obtained from all participants or their legal representatives. This study adhered to the International Ethical Guidelines and the World Medical Association Declaration of Helsinki. Approval for the study protocol was granted by the Independent Ethical Committee of the Old Town Hospital at the University Hospital Bratislava and the Faculty of Medicine, Comenius University in Bratislava, as well as the Independent Ethical Committee of the Bratislava Municipality.

### 2.2. Sample Collection and Processing

Peripheral venous blood samples from patients and control subjects were collected into tubes with ethylenediaminetetraacetic acid (EDTA) as an anticoagulant and subsequently processed by centrifugation (2000× *g* for 10 min) to obtain the buffy coat. Genomic DNA was isolated from the buffy coat samples using a modified phenol–chloroform extraction procedure [69]. After quantification with a NanoDrop1000 Spectrophotometer (Thermo Fisher Scientific, Waltham, MA, USA), all DNA samples were stored at −20 °C until genotyping analysis. Plasma samples were obtained from a subset of 156 patients, allocated into several microcentrifuge tubes, and immediately stored at −80 °C until further analysis.

### 2.3. SNP Selection and Genotyping

Eighteen common SNPs in the *ADIPOQ* gene were selected from the dbSNP database (https://www.ncbi.nlm.nih.gov/snp/, accessed on 9 January 2022) [70] after a thorough literature review. Their inclusion in the study was based on prior evidence of an association with circulating adiponectin levels in various population groups [33,35,36,37,38,39,40,48,51,55,56,58,59,60,64,71,72,73,74,75,76]. All SNPs had a minor allele frequency (MAF) of ≥5% in the European Caucasian population, according to the Allele Frequency Aggregator (ALFA) project database (https://www.ncbi.nlm.nih.gov/snp/docs/gsr/alfa/, accessed on 10 January 2022) [77]. Two of the SNPs included in the study are located in the 5′ flanking region (rs822387, rs860291), two in the gene promoter (rs17300539, rs266729), twelve in introns (rs182052, rs822393, rs822395, rs822396, rs7627128, rs2036373, rs17366568, rs17846866, rs1501299, rs2241767, rs3821799, rs3774261), and two in exons (rs2241766, rs1063539), as shown in Table 2.

*ADIPOQ* genotyping was performed using a polymerase chain reaction-restriction fragment length polymorphism method according to the modified protocols described elsewhere [71,75,78,79,80,81,82,83,84]. Detailed information on primer sequences, restriction enzymes, and specific product and fragment sizes is listed in Supplementary Table S1. For quality control, 10% of samples were randomly selected and genotyped in duplicate. Additionally, several cases of each genotype were confirmed by direct DNA sequencing using the BigDye^®^ Terminator v3.1 Cycle Sequencing Kit and the Applied Biosystems 3130xl Genetic Analyzer (Thermo Fisher Scientific, Waltham, MA, USA).

The carriage of the *APOE* ε4 allele, the single strongest genetic risk factor for AD, was determined by direct sequencing of exon 4 containing the rs429358:T>C and rs7412:C>T SNPs, as previously described [85]. The ε4 allele is defined by the presence of cytosine at both rs429358 and rs7412.

### 2.4. Measurement of Plasma Adiponectin Levels

To analyze the effects of individual *ADIPOQ* polymorphisms on adiponectin levels, we quantified total adiponectin in plasma samples from 156 LOAD patients (Supplementary Table S2) using a sandwich enzyme-linked immunosorbent assay and a commercially available HMW and Total Adiponectin ELISA kit (ALPCO, Salem, NH, USA).

### 2.5. Statistical Analyses

The comparison of categorical variables between study groups was performed by the χ^2^ test, while differences in continuous variables were assessed using the unpaired *t*-test with Welch correction (InStat version 3.10, GraphPad Software, San Diego, CA, USA). The relationship between two continuous variables was measured by Spearman’s rank correlation. The χ^2^ goodness-of-fit test with 1 degree of freedom was used to test for the possible departure of SNP genotypes from Hardy–Weinberg equilibrium (HWE), with a threshold of *p* ≤ 0.05 indicating deviation from HWE. Lewontin’s D’ and r^2^ values of linkage disequilibrium (LD) between SNP pairs were determined, and an LD plot was generated using the Haploview version 4.2 software [86]. Single-SNP and haplotype-based analyses of the association between *ADIPOQ* and LOAD risk were performed using the SNPStats version 0.96 web software [87], available at https://www.snpstats.net/ (accessed on 15 June 2023). Both the crude χ^2^ test and logistic regression analysis were employed, with the latter adjusted for potential confounding variables (age, sex, *APOE* ε4 carrier status, hypertension, T2DM, and BMI). Odds ratios (OR) with 95% confidence intervals (CI) and *p*-values were calculated under various inheritance models (allele, codominant, dominant, recessive, over-dominant, and log-additive). Correction for multiple comparisons was performed by the Bonferroni method, setting the corrected significance level at *p* ≤ 0.0028, obtained by dividing the standard significance *p*-value (0.05) by the number of tested SNPs (n = 18). Linear regression analysis was employed to examine the association between *ADIPOQ* SNPs and both the age of disease onset and plasma adiponectin levels. An uncorrected significance threshold of *p* ≤ 0.05 was applied to the latter parameter, considering the selection of SNPs was based on a priori information from published studies indicating their impact on adiponectin levels.

## 3. Results

### 3.1. Characteristics of Study Subjects

A total of 385 subjects with LOAD were enrolled in the case group, while 533 cognitively normal individuals comprised the control group. Table 1 presents the basic demographic and clinical characteristics of the study population, along with statistical comparisons between cases and controls. Significant differences were observed in mean age at examination, BMI, and MoCA score (*p* ˂ 0.0001). On the other hand, sex distribution was not significantly different between the study groups (*p* = 0.34), with females constituting the majority in both patients (63.1%) and controls (60.0%). T2DM was significantly more prevalent in patients with LOAD than in control subjects (*p* < 0.0001), while the difference was not quite significant for hypertension (*p* = 0.085). The AD risk allele *APOE* ε4 was present in at least one copy in over 50% of patients but only in 19% of controls (*p* < 0.0001), as outlined in Table 1.

### 3.2. Association of ADIPOQ Polymorphisms with LOAD Risk

**Figure 1:**
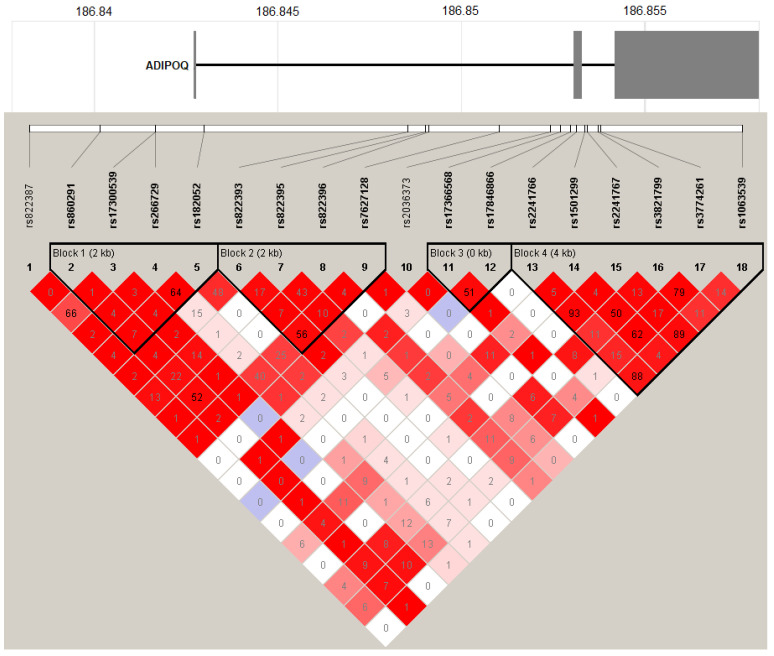
Gene map of *ADIPOQ* with the linkage disequilibrium (LD) plot for all SNPs genotyped in Slovak subjects. The upper part of the figure illustrates the structure of the *ADIPOQ* gene with the location of SNPs on a physical scale. LD plots and haplotype blocks were constructed with the Haploview version 4.2 software. The different colors and shadings in the LD plot represent the magnitude and significance of pairwise LD. Diamonds in the darkest shade indicate the highest LD (D’ = 1 and the logarithm of odds [LOD] ≥ 2) between two SNPs, with the color intensity decreasing with decreasing D’ value. White color indicates D’ value ˂ 1 with LOD score ˂ 2, while grey-blue color corresponds to D’ = 1 and LOD ˂ 2. The number within each diamond indicates the exact r^2^ pairwise correlation value multiplied by 100.

To assess the extent of LD across the *ADIPOQ* gene, we calculated pairwise D’ and r^2^ coefficients for the entire study population using Haploview 4.2 software. As illustrated in Figure 1 and detailed in Supplementary Table S3, the analysis of pairwise LD patterns for the 18 SNPs identified four distinct haplotype blocks encompassing the majority of the studied *ADIPOQ* variants. SNPs within individual haploblocks exhibited strong LD with one another, as indicated by pairwise D’ coefficients ranging from 0.88 to 1. However, they were weakly predictive of one another due to differing allele frequencies, as reflected in low to moderate pairwise r^2^ values ranging from 0.01 to 0.64. The only exception was observed in four SNP pairs within haploblock 4, which exhibited a high pairwise LD pattern as follows: rs2241766–rs2241767, rs2241766–rs1063539, rs2241767–rs1063539 (all with r^2^ > 0.88), and rs3821799–rs3774261 (r^2^ = 0.79).

The comparison of observed and expected genotype frequencies in the study cohorts demonstrated conformity to HWE for all investigated polymorphisms (Supplementary Table S4). Consequently, all 18 SNPs were included in subsequent analyses of association with LOAD risk. We employed both the crude χ^2^ test and multivariate logistic regression analysis, which adjusted for several potentially confounding factors, to examine the role of *ADIPOQ* SNPs in genetic susceptibility to LOAD. None of the inheritance models revealed a significant association between *ADIPOQ* variants and LOAD risk in the single-marker analyses (Table 3 and Supplementary Table S5). Similarly, no evidence of an association between *ADIPOQ* and disease risk was found in the haplotype-based analyses, as shown in Table 4.

To further evaluate the possible modifying effects of the *APOE* ε4 allele and sex on the association between *ADIPOQ* and LOAD risk, we also conducted statistical tests in cohorts stratified by these two covariates. These sub-analyses also did not yield any significant results, suggesting that *APOE* ε4 carrier status and sex do not influence the relationship between *ADIPOQ* variants and disease risk (Supplementary Tables S6–S9).

Linear regression analysis was used to assess the impact of *ADIPOQ* polymorphisms on the age of disease onset. Although some SNPs appeared to slightly accelerate (rs822387) or delay (rs860291) the onset of AD, none of the effects were statistically significant (Supplementary Table S10).

### 3.3. Association of ADIPOQ Polymorphisms with Plasma Adiponectin Levels

Total adiponectin concentrations were determined in plasma samples from 156 LOAD subjects (Supplementary Table S2) and compared between subgroups of patients stratified by sex, *APOE* ε4 carrier status, hypertension, T2DM, and treatment with acetylcholinesterase inhibitors (AChEI). As presented in Supplementary Table S11, mean adiponectin levels were significantly higher in females with LOAD compared to males (11.46 ± 5.81 μg/mL vs. 8.27 ± 3.71 μg/mL; *p* < 0.0001), while no differences could be found between subgroups of patients with or without hypertension (*p* = 0.34), T2DM (*p* = 0.80), or AChEI treatment (*p* = 0.67). Although patients with the *APOE* ε4 allele exhibited lower adiponectin levels than those without ε4, the difference did not reach statistical significance (*p* = 0.18). Spearman correlation results indicated a small but significant positive relationship of adiponectin levels with age (*r* = 0.19, *p* = 0.018) and a negative relationship with BMI (*r* = −0.17, *p* = 0.036).

Linear regression analysis controlled for potential confounders (age, sex, *APOE* ε4 carrier status, and BMI) revealed a significant association of two *ADIPOQ* SNPs (rs822395 and rs2036373) with alterations in total adiponectin levels in the plasma of LOAD patients. The −4041 A>C (rs822395) variant was associated with increased adiponectin under the dominant model (AC + CC vs. AA; *p* = 0.048), while the −657 T>G (rs2036373) SNP showed a similar effect in a TG vs. TT comparison (*p* = 0.043). Adiponectin concentrations also varied across different genotypes of several additional SNPs (rs822387, rs17300539, rs266729, rs182052, and rs822393); however, the *p*-values did not reach the threshold for statistical significance (Table 5).

## 4. Discussion

In this study, we conducted a case–control analysis to assess the association between eighteen *ADIPOQ* polymorphisms and LOAD risk in a cohort of 918 Slovak Caucasian individuals. Although we demonstrated the effect of several SNPs on circulating adiponectin, none of the variants studied were found to be associated with LOAD risk or to significantly affect the age of onset. Furthermore, neither sex nor the *APOE* ε4 allele had any effect on the association between *ADIPOQ* and AD.

Adiponectin is a 30 kDa adipocyte-derived protein that circulates in healthy subjects at relatively high concentrations (up to 30 μg/mL) [88]. Belonging to the soluble collagen superfamily, it shares structural similarities with complement factor C1q and tumor necrosis factor [19]. Newly synthesized adiponectin undergoes post-translational modifications and naturally self-associates to form trimers, hexamers, and high-molecular-weight (HMW) multimers. The HMW isoform constitutes the majority of circulating adiponectin and is considered the most biologically active form of adiponectin. The hormone exerts its actions by binding to specific receptors, namely AdipoR1, AdipoR2, and T-cadherin (CDH13), expressed in various tissues, including the liver, muscle, vascular endothelium, central nervous system, and others [3,19,88]. Despite its observed neuroprotective effects in cell-based and animal experiments [3,18,21,22,23,24,25], the role of adiponectin in the pathogenesis of AD in humans remains controversial, with some studies even suggesting its possible deleterious effects in amyloidogenesis [22,28,29,30,31].

*ADIPOQ* variants have been extensively studied for their association with circulating adiponectin, and numerous of them were identified as determinants of adiponectin levels in diverse population groups and metabolic syndrome-related phenotypes. In this study, we were able to extend the validity of this relationship to LOAD patients. Two of the SNPs, rs822395 (−4041 A>C) and rs2036373 (−657 T>G), showed a marginally significant association of their minor alleles with increased total plasma adiponectin after controlling for the confounding effects of age, sex, *APOE* ε4 allele, and BMI. Both SNPs are located in intron 1 at a distance of 3384 bp from each other, showing a low degree of correlation (LD r^2^ = 0.022), and belong to the less-studied *ADIPOQ* variants in relation to various phenotypes. Our results on rs822395 are in agreement with a meta-analysis of two general population cohorts consisting of 2355 subjects and one cohort of 967 subjects with T2DM [60] and a Coronary Artery Development in Young Adults (CARDIA) study in white men and women [73], both reporting significantly higher adiponectin in C allele carriers. Interestingly, the latter study also showed some indication of association for rs2036373, but the direction of the effect was opposite to that in our study. In contrast, other studies found no association between the two variants and adiponectin [33,55,71,74,76,89,90,91,92]. Additional SNPs in our study, located in the upstream region, promoter, or intron 1, showed a clear trend of adiponectin levels increasing (rs822387, rs17300539) or decreasing (rs266729, rs182052, rs822393) with each copy of the minor allele. Despite not quite reaching the significance threshold, these effects are consistent in magnitude and direction with those reported for the five variants (or their proxies) in several candidate genes and GWA studies on circulating adiponectin [33,36,37,38,40,41,42,44,46,47,48,49,50,51,54,55,56,57,59,60,61,62,64,71,72,73,74,75,91,92,93,94,95,96,97,98,99,100,101,102]. On the other hand, we failed to replicate previous, albeit often inconsistent, findings in the literature for several variants in haploblocks 3 and 4, particularly rs17366568 in intron 1 [35,36,45,47,51,57,59,60,76], rs2241766 in exon 2 [33,40,55,60,64,89,98,99,103], rs1501299, rs3821799, rs3774261 in intron 2 [33,35,36,37,40,51,54,55,57,59,60,64,71,75,76,89,90,91,99,100,103,104,105], and rs1063539 in the 3′-untranslated region (UTR) [36,55]. Several factors could account for the conflicting results across studies, including variations in sample sizes, disparities regarding the study group phenotype and analysis methodology, modifying effects of confounders and approaches to their control, gene–gene and gene–environment interactions, variations in the LD structure at the *ADIPOQ* locus between populations of different ethnic backgrounds, and others. Bioinformatics tools and in vitro functional analyses have suggested that *ADIPOQ* polymorphisms associated with alterations of adiponectin levels act through molecularly distinct mechanisms involved in controlling certain aspects of gene expression, such as the alteration of mRNA transcription, splicing, or stability [48,72,97,106,107]. Still, despite being the major gene for plasma adiponectin, robustly confirmed across various populations and ethnicities, *ADIPOQ* SNPs seem to explain only a relatively small proportion of the variance in circulating adiponectin levels, typically not exceeding 10% [36,55,58,59,60,62]. In contrast, non-genetic factors, such as age, sex, BMI, and clinical traits, appear to be more potent determinants of adiponectin concentrations [59,60,62]. In agreement with previous studies [33,37,76,100], we found that adiponectin levels were higher in females and correlated positively with age and negatively with BMI. Accordingly, these potential confounders were taken into account when analyzing the association of *ADIPOQ* SNPs with adiponectin levels and LOAD risk.

Although adiponectin has received attention in the context of AD, the role of its gene variants in genetic susceptibility to LOAD is still unclear. It could be hypothesized that polymorphisms capable of affecting circulating adiponectin levels would also contribute to LOAD risk. Two independent studies previously investigated the role of common *ADIPOQ* variants, namely, −11377 C>G (rs266729) and +276 G>T (rs1501299), in the predisposition to LOAD in the Chinese population, both reporting an association of the minor rs266729 G and 1501299 T alleles with increased LOAD risk [65,66]. The first of the two variants, rs266729, is located in the gene promoter region, where it was predicted to alter the sequence of one of four transcriptional stimulatory protein (SP1) binding sites [106]. The presence of the minor G allele resulted in a loss of SP1 binding to the promoter [106] and lower transcriptional activity [97]. Moreover, when analyzed together with two other promoter variants, rs16861194 and rs17300539, minor alleles of all three *ADIPOQ* SNPs led to a complete loss of promoter activity [72]. In humans, the rs266729 G allele has been relatively consistently shown to reduce adiponectin levels in several studies [33,36,40,41,64,72,75,92,93,94,96,97,98,99,100,101], including ours. In contrast, rs1501299 in intron 2 seems to have an opposite effect on adiponectin compared to rs266729, with the minor T allele increasing adiponectin levels, as shown by previous meta-analyses of candidate gene studies [54,64] and GWAS [41]. However, rs1501299 is likely not a true functional variant [97] but may act as a proxy SNP in LD with a different variant shown to influence adiponectin levels, e.g., rs6444175 [38,56,60,74] and rs7639352 [51,74] in the 3′ downstream region, or rs6773957 [35,56,57,58] and rs56354395 [33,103] in the 3′ UTR. The observed association of both adiponectin-decreasing rs266729 G and adiponectin-increasing rs1501299 T alleles with a higher risk of LOAD in the Chinese studies seems contradictory, but it may reflect the complex nature of *ADIPOQ* gene architecture and the effects of its variants. Adding more ambiguity, our study did not reveal any significant differences in allele and genotype distribution of rs266729 and rs1501299 between the patient and control groups, thus failing to replicate the association of the two variants with LOAD risk in the Slovak Caucasian population. The lack of association was also evident after controlling for potential confounders, including known or presumed AD risk factors such as age, sex, the *APOE* ε4 allele, BMI, hypertension, and T2DM. To increase gene coverage, we also analyzed an additional 16 variants across the *ADIPOQ* gene and its upstream region but again found no evidence of association with disease risk in either single-SNP or haplotype-based analyses. In addition to adjusting for *APOE* ε4 and sex, we also performed subgroup analyses in cohorts stratified according to these two parameters. The lack of any significant results from them led us to conclude that the *APOE* ε4 allele and sex have no significant impact on the association between *ADIPOQ* SNPs and LOAD risk.

Overall, our results were not consistent with the previous reports on the role of *ADIPOQ* in the predisposition to LOAD in the Chinese population [65,66], suggesting possible inter-ethnic differences in the genetic background of the disease. Based on our findings, we posit that despite the obvious effects of *ADIPOQ* SNPs on circulating adiponectin, this impact does not translate into a significantly increased risk of developing LOAD. This conclusion aligns with the results of a recent MR study that explored the causality between circulating adiponectin and AD risk, finding no association between genetically predicted adiponectin levels and AD [108]. The 14 SNPs used as instrumental variables in the MR analysis were selected from a 2012 GWAS meta-analysis from the ADIPOGen consortium [41] and included four variants within or in proximity to *ADIPOQ*. Considering these findings, it seems plausible that elevated adiponectin observed in AD patients [27] is a biomarker or a bystander rather than a causal risk factor. Alternatively, it may represent a compensatory effort against neurodegeneration, aiming to aid the clearance of Aβ aggregates, e.g., by controlling the expression of *ABCA1*, an important promoter of efferocytosis [109].

Some limitations of the current study need to be acknowledged. First, while its statistical power to replicate the findings of two previous studies [65,66] was over 90% (as assessed by the Genetic Association Study Power Calculator available at https://csg.sph.umich.edu/abecasis/gas_power_calculator/index.html, accessed on 10 January 2022), this power inevitably decreased when weaker effect sizes (OR < 1.10) and a corrected significance level of 0.0028 were considered. Hence, we are aware that false-negative results cannot be completely ruled out, and additional analyses in independent populations are warranted to validate our findings. A similar limitation related to sample size also applied to the linear regression analysis of the association between *ADIPOQ* and plasma adiponectin in LOAD patients and could be one of the reasons why we failed to detect a significant effect of some SNPs on adiponectin levels. Second, although we made an attempt to adjust our analyses for several important parameters related to AD risk, there are still other possible confounders that could not be accounted for, such as educational attainment, dyslipidemia, other diseases, smoking, drinking, and other lifestyle factors. This, along with differences in inclusion and exclusion criteria and the modifying effects of additional genetic and environmental factors, may be another source of heterogeneity in the results of studies. Third, our study exclusively focused on SNPs with MAF ≥ 5%. Apart from these common polymorphisms with relatively subtle effects, the *ADIPOQ* gene also harbors several low-frequency variants (MAF < 5%) previously associated with alterations in circulating adiponectin levels, such as rs17366653 in intron 1 [48,49,50,58] and rs17366743 in exon 3 [58,75,76]. Hence, these variants could be considered potential candidates for future studies on the association between *ADIPOQ* and AD risk. Moreover, numerous rare nonsynonymous missense or nonsense variants have been identified in exons 2 and 3, with several predicted to be damaging or deleterious by in silico analyses. Such gene mutations altering the amino acid composition may impact protein structure, multimerization, function, subcellular localization, or secretion, ultimately affecting various biological processes associated with adiponectin [110]. Some of these rare variants, such as rs62625753 (Gly90Ser), rs199646033 (Gly84Arg), and rs143606172 (Arg55His), are associated with hypoadiponectinemia due to changes in the amino acid sequence resulting in the inability to form HMW multimers. Others, such as rs185847354 (Ile164Thr) and rs121917815 (Arg112Cys), disrupt the assembly into low-molecular-weight trimers [33,47,48,62,74,110,111,112,113,114]. Interestingly, the rs62625753 (c.268G>A; Gly90Ser) mutation in exon 3 was recently shown to produce abnormal aggregation of tau proteins and contribute to cognitive degeneration, suggesting a functional impact for AD [115]. Hence, large-scale studies focusing on low-frequency and rare exon variants may have the potential to identify causal *ADIPOQ* mutations with larger pathogenic effects in a small subset of AD patients, eventually helping to explain a portion of the so-called missing heritability.

## 5. Conclusions

While adiponectin has emerged as a potential contributor to the development of LOAD due to its complex effects on energy metabolism and neurological processes, this study failed to find any significant involvement of its gene variants in susceptibility to the disease. Although we confirmed the influence of some of the SNPs on circulating adiponectin, this impact does not appear to modulate the risk of developing LOAD. Further studies are required to uncover whether rare *ADIPOQ* variants or genes coding for other molecules involved in adiponectin signaling contribute to LOAD risk.

## Figures and Tables

**Table 1:** Demographic and clinical characteristics of LOAD patients and control subjects.

Parameter	LOAD (n = 385)	Controls (n = 533)	*p*-Value
Age at examination (years)	77.73 ± 6.36	76.35 ± 7.68	0.0031
Age of onset (years)	75.06 ± 6.39	-	-
Sex (female/male)	243 (63.12%)/142 (36.88%)	320 (60.04%)/213 (39.96%)	0.34
MoCA score	16.15 ± 5.82	27.53 ± 1.40	<0.0001
BMI (kg/m^2^)	26.57 ± 4.53	27.77 ± 3.99	<0.0001
Hypertension (yes/no)	277 (71.95%)/108 (28.05%)	355 (66.60%)/178 (33.40%)	0.085
T2DM (yes/no)	93 (24.16%)/292 (75.84%)	72 (13.51%)/461 (86.49%)	<0.0001
*APOE* ε4 positivity (yes/no)	195 (50.65%)/190 (49.35%)	101 (18.95%)/432 (81.05%)	<0.0001

Data are presented as the mean ± standard deviation or as absolute values with % in parentheses. *APOE*: apolipoprotein E gene; BMI: body mass index; LOAD: late-onset Alzheimer’s disease; MoCA: Montreal Cognitive Assessment; T2DM: type 2 diabetes mellitus.

**Table 2:** List of investigated *ADIPOQ* polymorphisms.

dbSNP	Alleles ^a^	Position ^b^	RefSeqGene	Gene Region	Aliases
rs822387	T>C	chr3:186838248	NG_021140.1:g.575T>C	5′-flanking region	−14,811 T/C
rs860291	C>T	chr3:186840168	NG_021140.1:g.2495T>C	5′-flanking region	−12,823 C/T; −12,891 C/T
rs17300539	G>A	chr3:186841671	NG_021140.1:g.3998G>A	Promoter	−11,388G/A; −11,391 G/A
rs266729	C>G	chr3:186841685	NG_021140.1:g.4012C>G	Promoter	−11,377 C/G; −11,365 C/G
rs182052	G>A	chr3:186842993	NG_021140.1:g.5320G>A	Intron 1	−10,066 G/A; −10,068 G/A
rs822393	C>T	chr3:186848537	NG_021140.1:g.10864C>T	Intron 1	−4522 C/T
rs822395	A>C	chr3:186849018	NG_021140.1:g.11345C>A	Intron 1	−4034 A/C; −4041 A/C
rs822396	A>G	chr3:186849088	NG_021140.1:g.11415G>A	Intron 1	−3964 A/G; −3971 A/G
rs7627128	C>A	chr3:186851010	NG_021140.1:g.13337C>A	Intron 1	−2049 C/A
rs2036373	T>G	chr3:186852402	NG_021140.1:g.14729T>G	Intron 1	−657 T/G
rs17366568	G>A	chr3:186852664	NG_021140.1:g.14991G>A	Intron 1	−395 G/A
rs17846866	T>G	chr3:186852957	NG_021140.1:g.15284T>G	Intron 1	−102 T/G
rs2241766	T>G	chr3:186853103	NG_021140.1:g.15430T>G	Exon 2	+45 T/G; Gly15Gly; T94G
rs1501299	G>T	chr3:186853334	NG_021140.1:g.15661G>T	Intron 2	+276 G/T
rs2241767	A>G	chr3:186853407	NG_021140.1:g.15734A>G	Intron 2	+349 A/G
rs3821799	C>T	chr3:186853697	NG_021140.1:g.16024T>C	Intron 2	+639 C/T
rs3774261	G>A	chr3:186853770	NG_021140.1:g.16097A>G	Intron 2	+712 G/A
rs1063539	G>C	chr3:186857603	NG_021140.1:g.19930G>C	Exon 3/3′ UTR	+4545 G/C

^a^ Alleles are listed as major allele>minor allele. ^b^ SNP position according to Genome Reference Consortium Human Build 38 patch release 14 (GRCh38.p14). *ADIPOQ*: adiponectin gene; SNP: single nucleotide polymorphism; UTR: untranslated region.

**Table 3:** Association between *ADIPOQ* polymorphisms and LOAD risk in the whole study population.

*ADIPOQ* SNPs	LOAD (n = 385)		Controls (n = 533)		Logistic Regression Analysis (Log-Additive Model)	
	Genotypes ^a^	MAF	Genotypes ^a^	MAF	*p*-Value	OR (95% CI)
rs822387 T>C	336/46/3	0.0675	463/65/5	0.0704	0.64	0.91 (0.62–1.34)
rs860291 C>T	301/80/4	0.1143	425/105/3	0.1041	0.54	1.11 (0.80–1.53)
rs17300539 G>A	335/49/1	0.0662	457/70/6	0.0769	0.32	0.82 (0.56–1.21)
rs266729 C>G	206/147/32	0.2740	279/211/43	0.2786	0.66	0.95 (0.76–1.18)
rs182052 G>A	151/175/59	0.3805	212/252/69	0.3659	0.72	1.04 (0.85–1.27)
rs822393 C>T	213/139/33	0.2662	296/205/32	0.2523	0.36	1.11 (0.89–1.39)
rs822395 A>C	170/167/48	0.3416	240/229/64	0.3349	0.86	0.98 (0.80–1.21)
rs822396 A>G	256/115/14	0.1857	358/159/16	0.1792	0.80	0.97 (0.75–1.25)
rs7627128 C>A	269/106/10	0.1636	363/153/17	0.1754	0.59	0.93 (0.71–1.21)
rs2036373 T>G	343/42/0	0.0545	475/57/1	0.0553	0.62	1.12 (0.72–1.75)
rs17366568 G>A	302/79/4	0.1130	401/127/5	0.1285	0.47	0.89 (0.65–1.22)
rs17846866 T>G	329/56/0	0.0727	464/67/2	0.0666	0.48	1.15 (0.77–1.72)
rs2241766 T>G	304/77/4	0.1104	427/100/6	0.1051	0.66	1.08 (0.78–1.48)
rs1501299 G>T	184/167/34	0.3052	270/215/48	0.2917	0.24	1.14 (0.92–1.41)
rs2241767 A>G	306/77/2	0.1052	430/97/6	0.1022	0.79	1.05 (0.75–1.46)
rs3821799 C>T	112/191/82	0.4610	168/251/114	0.4493	0.28	1.12 (0.92–1.36)
rs3774261 G>A	134/186/65	0.4104	202/242/89	0.3940	0.21	1.14 (0.93–1.39)
rs1063539 G>C	302/80/3	0.1117	423/104/6	0.1088	0.84	1.03 (0.75–1.43)

^a^ Genotype numbers are presented as follows: major allele homozygotes/heterozygotes/minor allele homozygotes. Logistic regression analysis was adjusted for age, sex, *APOE* ε4 carrier status, hypertension, type 2 diabetes mellitus, and body mass index. *P*, OR, and 95% CI values are shown for the log-additive model. Values obtained by crude analysis and under other genetic models are presented in Supplementary Table S5. *ADIPOQ*: adiponectin gene; CI: confidence interval; LOAD: late-onset Alzheimer’s disease; MAF: minor allele frequency; OR: odds ratio; SNP: single nucleotide polymorphism.

**Table 4:** Association between *ADIPOQ* haplotypes and LOAD risk in the whole study population.

*ADIPOQ* Haplotypes	LOAD (n = 385)	Controls (n = 533)	Logistic Regression Analysis	
	EHF	EHF	*p*-Value	OR (95% CI)
Block 1 (rs860291–rs17300539–rs266729–rs182052)				
CGCG	0.4390	0.4531		Reference
CGGA	0.2740	0.2786	0.87	0.98 (0.77–1.24)
TGCG	0.1143	0.1041	0.55	1.11 (0.79–1.56)
CGCA	0.1065	0.0872	0.25	1.23 (0.87–1.74)
CACG	0.0662	0.0769	0.43	0.85 (0.57–1.27)
Block 2 (rs822393–rs822395–rs822396–rs7627128)				
CAAC	0.3926	0.4116		Reference
CCGC	0.1857	0.1781	0.87	1.02 (0.77–1.36)
TAAA	0.1622	0.1713	0.95	0.99 (0.74–1.32)
CCAC	0.1540	0.1549	0.82	1.04 (0.77–1.40)
TAAC	0.1022	0.0781	0.062	1.42 (0.98–2.05)
Block 3 (rs17366568–rs17846866)				
GT	0.8857	0.8705		Reference
AG	0.0714	0.0656	0.57	1.12 (0.75–1.68)
AT	0.0416	0.0629	0.083	0.66 (0.42–1.05)
Block 4 (rs2241766–rs1501299–rs2241767–rs3821799–rs3774261–rs1063539)				
TGACGG	0.5237	0.5413		Reference
TTATAG	0.2997	0.2917	0.24	1.15 (0.91–1.44)
GGGTAC	0.0993	0.1004	0.69	1.07 (0.76–1.51)
TGATGG	0.0537	0.0553	0.75	1.08 (0.68–1.69)

Haplotype frequencies were estimated using the expectation–maximization algorithm implemented in the SNPStats version 0.96 software. Only haplotypes with a frequency > 5% are shown. Logistic regression analysis was adjusted for age, sex, *APOE* ε4 carrier status, hypertension, type 2 diabetes mellitus, and body mass index. *ADIPOQ*: adiponectin gene; CI: confidence interval; EHF: estimated haplotype frequency; LOAD: late-onset Alzheimer’s disease; OR: odds ratio.

**Table 5:** Association between *ADIPOQ* polymorphisms and plasma adiponectin levels in 156 LOAD patients.

*ADIPOQ* SNPs	Adiponectin Levels (μg/mL) ^a^			Genetic Model	*p*_adj_-Value ^b^	Δ (95% CI) ^b^
	1/1	1/2	2/2			
rs822387 T>C	10.14 ± 5.36 (136)	11.95 ± 5.86 (18)	13.39 ± 0.46 (2)	A	0.14	1.61 (−0.50–3.72)
rs860291 C>T	10.18 ± 5.11 (126)	11.23 ± 6.68 (29)	12.41 ± 0.00 (1)	A	0.50	0.67 (−1.29–2.64)
rs17300539 G>A	10.19 ± 5.39 (138)	11.89 ± 5.65 (17)	13.07 ± 0.00 (1)	A	0.20	1.55 (−0.80–3.89)
rs266729 C>G	11.05 ± 5.72 (80)	10.04 ± 5.16 (62)	8.18 ± 4.08 (14)	R	0.12	−2.26 (−5.09–0.56)
rs182052 G>A	11.66 ± 5.76 (55)	9.79 ± 5.48 (73)	9.46 ± 4.05 (28)	D	0.075	−1.56 (−3.26–0.14)
rs822393 C>T	11.09 ± 5.64 (81)	9.72 ± 5.44 (57)	9.33 ± 3.86 (18)	D	0.10	−1.36 (−2.97–0.26)
rs822395 A>C	9.68 ± 4.95 (75)	10.89 ± 5.68 (64)	11.65 ± 6.16 (17)	D	0.048	1.64 (0.03–3.25)
rs822396 A>G	10.30 ± 5.13 (104)	10.55 ± 6.15 (47)	10.90 ± 4.71 (5)	D	0.83	0.19 (−1.54–1.91)
rs7627128 C>A	10.77 ± 5.56 (107)	9.66 ± 5.25 (44)	8.72 ± 2.12 (5)	A	0.33	−0.76 (−2.27–0.75)
rs2036373 T>G	10.07 ± 5.17 (140)	13.18 ± 6.76 (16)	-	-	0.043	2.80 (0.12–5.49)
rs17366568 G>A	10.27 ± 5.19 (125)	10.97 ± 6.45 (28)	10.06 ± 5.51 (3)	D	0.20	1.36 (−0.70–3.43)
rs17846866 T>G	10.28 ± 5.20 (135)	11.08 ± 6.70 (21)	-	-	0.18	1.65 (−0.76–4.07)
rs2241766 T>G	10.56 ± 5.25 (127)	9.24 ± 5.79 (28)	21.38 ± 0.00 (1)	O	0.18	−1.45 (−3.58–0.67)
rs1501299 G>T	10.41 ± 5.06 (73)	10.30 ± 5.82 (69)	10.72 ± 5.43 (14)	D	0.99	−0.01 (−1.66–1.65)
rs2241767 A>G	10.54 ± 5.29 (125)	9.80 ± 5.94 (31)	-	-	0.35	−0.98 (−3.03–1.07)
rs3821799 C>T	10.42 ± 4.63 (47)	10.50 ± 5.86 (76)	10.10 ± 5.52 (33)	R	0.60	−0.54 (−2.54–1.46)
rs3774261 G>A	10.26 ± 4.47 (54)	10.78 ± 6.11 (76)	9.52 ± 5.08 (26)	R	0.22	−1.38 (−3.56–0.80)
rs1063539 G>C	10.54 ± 5.29 (125)	9.41 ± 5.63 (30)	21.38 ± 0.00 (1)	O	0.20	−1.36 (−3.43–0.71)

^a^ Adiponectin levels are presented as the mean ± standard deviation, with the absolute genotype numbers shown in parentheses. ^b^ Linear regression analysis was adjusted for age, sex, *APOE* ε4 carrier status, and body mass index. Differences (Δ) in adiponectin levels, 95% CI, and *p*-values are shown for the best genetic model with the lowest Akaike information (AIC). 1/1: major allele homozygotes; 1/2: heterozygotes; 2/2: minor allele homozygotes; A: log-additive; *ADIPOQ*: adiponectin gene; D: dominant; LOAD: late-onset Alzheimer’s disease; O: over-dominant; R: recessive; SNP: single nucleotide polymorphism.

## Data Availability

The data presented in this study are available on request from the corresponding author.

## Supplementary Materials

The following supporting information can be downloaded at: https://www.mdpi.com/article/10.3390/life14030346/s1, Table S1: Primer sequences and PCR-RFLP genotyping conditions for *ADIPOQ* SNPs; Table S2: Demographic and clinical characteristics of LOAD patients included in the analysis of the association between *ADIPOQ* SNPs and plasma adiponectin levels; Table S3: Linkage disequilibrium data (D’, r^2^) of the *ADIPOQ* SNPs observed in the overall study population; Table S4: Hardy–Weinberg equilibrium values in patient and control populations; Table S5: Association between *ADIPOQ* SNPs and LOAD risk in the whole study population; Table S6: Association between *ADIPOQ* polymorphisms and LOAD risk in the *APOE* ε4-positive subpopulation; Table S7: Association between *ADIPOQ* polymorphisms and LOAD risk in the *APOE* ε4-negative subpopulation; Table S8: Association between *ADIPOQ* polymorphisms and LOAD risk in female subjects; Table S9: Association between *ADIPOQ* polymorphisms and LOAD risk in male subjects; Table S10: Association between *ADIPOQ* polymorphisms and age of AD onset in LOAD patients; Table S11: Plasma adiponectin levels in different subgroups of patients with LOAD.
