# Supplementary material for: Adiponectin Gene Polymorphisms: A Case–Control Study on Their Role in Late-Onset Alzheimer’s Disease Risk
Source: Life (Basel). 2024 Mar 7;14(3):346. doi: 10.3390/life14030346 (PMC10971943; doi:10.3390/life14030346)
Supplement: Supplementary file 1 [file life-14-00346-s001.zip › Supplementary Table S1.pdf]

Supplementary Table S1. Primer sequences and PCR-RFLP genotyping conditions for *ADIPOQ* SNPs.

SNP	Primer Sequence	Ta	Restriction Enzyme	Fragment Length (bp)
rs822387	F: 5′-ACTGTGCCTGGCCTAGTGTT-3′<br>R: 5′-TGCCCACAGTAGGAAGTGAA-3′	54°C	XbaI	T: 294 + 286<br>C: 582
rs860291	F: 5′-CCACCTTGTGCAATAGCTACGGC-3′<br>R: 5′-GGGCAACAGAGCGAGAATCCAT-3′	60°C	BsuRI	C: 81 + 22<br>T: 103
rs17300539	F: 5′-ACTTGCCCTGCCTCTGTCTG-3′<br>R: 5′-GCAAGTTAAAAAGGCCCTGA-3′	58°C	MspI	G: 396 + 121<br>A: 517
rs266729	F: 5′-ACTTGCCCTGCCTCTGTCTG-3′<br>R: 5′-GCAAGTTAAAAAGGCCCTGA-3′	58°C	HhaI	C: 517<br>G: 380 + 137
rs182052	F: 5′-TCTCCCGTCCGTAGTAGTGT-3′<br>R: 5′-TAGGTATCCTGCTACAGAGCCAACT-3′	58°C	BglI	G: 177 + 22<br>A: 199
rs822393	F: 5′-TTCCAAATCGGTGAGCTTTC-3′<br>R: 5′-AGGGCTTTGGGAAGATTAGG-3′	52°C	HinfI	C: 137 + 65<br>T: 202
rs822395	F: 5′-TGATCGCACCTATTAGTGGAGGAAT-3′<br>R: 5′-TCCAGAATATGTGAAAGCCCCAGAG-3′	58°C	HinfI	A: 208<br>C: 186 + 22
rs822396	F: 5′-CAGGCTGATCGCACCTATTA-3′<br>R: 5′-CATCCTTTTCTGCTGGGAAA-3′	58°C	Tru1I	A: 147 + 99<br>G: 246
rs7627128	F: 5′-GAGTTGGATGTGCCACGTGAAGAGGGTAGTATAGTA-3′<br>R: 5′-TGAGGGATGGAAGAAAGACCGTGAT-3′	62°C	RsaI	C: 94 + 35<br>A: 129
rs2036373	F: 5′-GAGAGACACATCATCCCGTG-3′<br>R: 5′-GGGTCCTGAAAGCTGAATAGAG-3′	57°C	Alw44I	T: 106<br>G: 84 + 22
rs17366568	F: 5′-CCAGGAGAGGGGTAGAGGAAG-3′<br>R: 5′-TAATTTTCCCATGTCTGAGCA-3′	58°C	Tru1I	G: 237<br>A: 124 + 113
rs17846866	F: 5′-GCTAAGTATTACAGATTTCAGGGCAG-3′<br>R: 5′-CAGCCATGGAGAGACAGACCC-3′	60°C	HinfI	T: 107 + 55 + 19<br>G: 126 + 55
rs2241766	F: 5′-CTGAGATGGACGGAGTCCTTT-3′<br>R: 5′-CCAAATCACTTCAGGTTGCTT-3′	60°C	SmaI	T: 456<br>G: 316 + 140
rs1501299	F: 5′-CTGAGATGGACGGAGTCCTTT-3′<br>R: 5′-CCAAATCACTTCAGGTTGCTT-3′	60°C	Mva1269I	G: 374 + 82<br>T: 456
rs2241767	F: 5′-GGTGAGAAGGGTGAGAAAGGAG-3′<br>R: 5′-TACTGGGAATAGGGATGAGGG-3′	60°C	Eco81I	A: 292<br>G: 159 + 133
rs3821799	F: 5′-CCTTTCCCATCTTCACCCTCA-3′<br>R: 5′-TCATTCTTCATCAGGTCCACG-3′	60°C	Eco91I	C: 191 + 107<br>T: 298
rs3774261	F: 5′-TGGCATTCAACCACATTTAC-3′<br>R: 5′-AAGCCTTCATTCTTCATCAG-3′	52°C	RsaI	G: 184 + 33<br>A: 217
rs1063539	F: 5′-TGGCTATGCTCACAGTCTCAC-3′<br>R: 5′-ACTTCAAAGCATCACAGGACC-3′	60°C	HinfI	G: 119 + 79<br>C: 198

The underlined bases indicate artificial substitutions that were introduced to create restriction site for endonuclease. bp: base pair; F: forward; PCR: polymerase chain reaction; R: reverse; RFLP: restriction fragment length polymorphism; SNP: single nucleotide polymorphism; Ta: annealing temperature.
