# Supplementary material for: Adiponectin Gene Polymorphisms: A Case–Control Study on Their Role in Late-Onset Alzheimer’s Disease Risk
Source: Life (Basel). 2024 Mar 7;14(3):346. doi: 10.3390/life14030346 (PMC10971943; doi:10.3390/life14030346)
Supplement: Supplementary file 1 [file life-14-00346-s001.zip › Supplementary Table S10.pdf]

**Supplementary Table S10.** Association between *ADIPOQ* polymorphisms and age of AD onset in LOAD patients.

| *ADIPOQ* SNPs | Age of Disease Onset (Years)[a] | | | Genetic Model | $p_{adj}$-Value[b] | Δ (95% CI)[b] |
|---|---|---|---|---|---|---|
| | 1/1 | 1/2 | 2/2 | | | |
| rs822387 T>C | 75.26 ± 6.29 | 73.91 ± 7.05 | 70.67 ± 6.03 | Log-additive | 0.13 | -1.32 (-3.03 – 0.39) |
| rs860291 C>T | 74.80 ± 6.29 | 75.95 ± 6.74 | 77.25 ± 7.04 | Log-additive | 0.12 | 1.12 (-0.28 – 2.53) |
| rs17300539 G>A | 75.10 ± 6.33 | 74.90 ± 6.89 | 70.00 ± 0.00 | Log-additive | 0.80 | -0.24 (-2.04 – 1.56) |
| rs266729 C>G | 75.25 ± 6.57 | 74.76 ± 6.37 | 75.28 ± 5.44 | Over-dominant | 0.75 | -0.21 (-1.50 – 1.08) |
| rs182052 G>A | 75.03 ± 6.95 | 74.89 ± 6.02 | 75.66 ± 6.04 | Recessive | 0.47 | 0.64 (-1.10 – 2.37) |
| rs822393 C>T | 74.93 ± 6.78 | 75.09 ± 5.95 | 75.76 ± 5.72 | Recessive | 0.50 | 0.77 (-1.46 – 3.00) |
| rs822395 A>C | 75.19 ± 5.96 | 74.99 ± 6.74 | 74.85 ± 6.74 | Log-additive | 0.91 | -0.05 (-0.97 – 0.87) |
| rs822396 A>G | 74.90 ± 6.36 | 75.24 ± 6.65 | 76.50 ± 4.80 | Log-additive | 0.28 | 0.63 (-0.50 – 1.76) |
| rs7627128 C>A | 75.22 ± 6.57 | 74.70 ± 6.02 | 74.70 ± 5.81 | Log-additive | 0.48 | -0.43 (-1.62 – 0.77) |
| rs2036373 T>G | 75.14 ± 6.30 | 74.43 ± 7.19 | - | - | 0.36 | -0.93 (-2.94 – 1.07) |
| rs17366568 G>A | 75.33 ± 6.12 | 74.03 ± 7.07 | 75.00 ± 11.66 | Over-dominant | 0.15 | -1.15 (-2.69 – 0.39) |
| rs17846866 T>G | 75.18 ± 6.27 | 74.36 ± 7.08 | - | - | 0.41 | -0.74 (-2.51 – 1.03) |
| rs2241766 T>G | 74.99 ± 6.45 | 75.23 ± 6.27 | 77.25 ± 5.06 | Recessive | 0.58 | 1.75 (-4.41 – 7.92) |
| rs1501299 G>T | 75.19 ± 6.46 | 74.71 ± 6.33 | 76.09 ± 6.38 | Over-dominant | 0.33 | -0.63 (-1.89 – 0.64) |
| rs2241767 A>G | 74.97 ± 6.48 | 75.49 ± 6.14 | 73.00 ± 0.00 | Over-dominant | 0.52 | 0.51 (-1.05 – 2.07) |
| rs3821799 C>T | 74.57 ± 6.07 | 75.42 ± 6.66 | 74.89 ± 6.21 | Over-dominant | 0.20 | 0.83 (-0.43 – 2.08) |
| rs3774261 G>A | 74.65 ± 6.49 | 75.51 ± 6.44 | 74.63 ± 6.06 | Over-dominant | 0.20 | 0.82 (-0.43 – 2.07) |
| rs1063539 G>C | 74.83 ± 6.43 | 75.88 ± 6.27 | 76.33 ± 5.77 | Log-additive | 0.23 | 0.88 (-0.56 – 2.31) |

[a]Age of onset is expressed as mean ± standard deviation. [b]Linear regression analysis was adjusted for sex, *APOE* ε4 carrier status, hypertension, type 2 diabetes mellitus, and body mass index. Difference (**Δ**) in age of onset, 95% CI and *p*-values are shown for the best genetic model with the lowest Akaike information (AIC). 1/1: major allele homozygotes; 1/2: heterozygotes; 2/2: minor allele homozygotes; *ADIPOQ*: adiponectin gene; *APOE*: apolipoprotein E gene; LOAD: late-onset Alzheimer's disease; SNP: single nucleotide polymorphism.
