# Supplementary material for: Adiponectin Gene Polymorphisms: A Case–Control Study on Their Role in Late-Onset Alzheimer’s Disease Risk
Source: Life (Basel). 2024 Mar 7;14(3):346. doi: 10.3390/life14030346 (PMC10971943; doi:10.3390/life14030346)
Supplement: Supplementary file 1 [file life-14-00346-s001.zip › Supplementary Table S11.pdf]

Supplementary Table S11. Plasma adiponectin levels in different subgroups of patients with LOAD.

Patient Group	Adiponectin Levels (µg/ml)	*p*-Value
Total LOAD patient population (n = 156)	10.39 ± 5.41	-
Female patients (n = 104)	11.46 ± 5.81	<0.0001
Male patients (n = 52)	8.27 ± 3.71	
Patients with *APOE* ε4 (n = 80)	9.82 ± 4.70	0.18
Patients without *APOE* ε4 (n = 76)	10.99 ± 6.04	
Patients with hypertension (n = 115)	10.62 ± 5.61	0.34
Patients without hypertension (n = 41)	9.74 ± 4.81	
Patients with T2DM (n = 38)	10.17 ± 6.20	0.80
Patients without T2DM (n = 118)	10.46 ± 5.16	
Patients with AChEI treatment (n = 87)	10.55 ± 5.64	0.67
Patients without AChEI treatment (n = 69)	10.18 ± 5.14	

Adiponectin levels are expressed as mean ± standard deviation. Differences between the LOAD subgroups were analyzed by the unpaired t test with Welch correction. AChEI: acetylcholinesterase inhibitor; *ADIPOQ*: adiponectin gene; *APOE*: apolipoprotein E gene; LOAD: late-onset Alzheimer's disease; T2DM: type 2 diabetes mellitus.
