# Supplementary material for: Adiponectin Gene Polymorphisms: A Case–Control Study on Their Role in Late-Onset Alzheimer’s Disease Risk
Source: Life (Basel). 2024 Mar 7;14(3):346. doi: 10.3390/life14030346 (PMC10971943; doi:10.3390/life14030346)
Supplement: Supplementary file 1 [file life-14-00346-s001.zip › Supplementary Table S2.pdf]

Supplementary Table S2. Demographic and clinical characteristics of LOAD patients included in the analysis of association between *ADIPOQ* SNPs and plasma adiponectin levels.

**Parameter**	**LOAD** (n = 156)
Age at examination (years)	79.01 ± 5.77
Age of onset (years)	75.72 ± 6.13
Sex (female/male)	104 (66.67%)/52 (33.33%)
MoCA score	16.83 ± 5.37
BMI (kg/m^2)	26.94 ± 4.68
Hypertension (yes/no)	115 (73.72%)/41 (26.28%)
T2DM	38 (24.36%)/118 (75.64%)
AChEI treatment	87 (55.77%)/69 (44.23%)
APOE ε4 positivity	80 (51.28%)/76 (48.72%)

Data are expressed as mean ± standard deviation or as absolute values with percentages in parentheses. AChEI: acetylcholinesterase inhibitor; *ADIPOQ*: adiponectin gene; *APOE*: apolipoprotein E gene; BMI: body mass index; LOAD: late-onset Alzheimer's disease; MoCA: Montreal Cognitive Assessment; T2DM: type 2 diabetes mellitus.
