# Supplementary material for: Adiponectin Gene Polymorphisms: A Case–Control Study on Their Role in Late-Onset Alzheimer’s Disease Risk
Source: Life (Basel). 2024 Mar 7;14(3):346. doi: 10.3390/life14030346 (PMC10971943; doi:10.3390/life14030346)
Supplement: Supplementary file 1 [file life-14-00346-s001.zip › Supplementary Table S3.pdf]

Supplementary Table S3. Linkage disequilibrium data (D', r²) of the *ADIPOQ* SNPs observed in the overall study population.

	rs822387	rs860291	rs17300539	rs266729	rs182052	rs822393	rs822395	rs822396	rs7627128	rs2036373	rs17366568	rs17846866	rs2241766	rs1501299	rs2241767	rs3821799	rs3774261	rs1063539
rs822387	-	1.0	0.838	1.0	1.0	1.0	0.969	1.0	1.0	0.675	0.266	1.0	0.031	0.609	0.03	0.702	0.768	0.007
rs860291	0.009	-	1.0	1.0	1.0	1.0	0.97	0.98	1.0	0.587	1.0	1.0	1.0	0.954	1.0	0.959	0.974	1.0
rs17300539	0.668	0.01	-	1.0	1.0	1.0	0.97	1.0	1.0	1.0	1.0	1.0	0.027	0.795	0.036	0.937	0.964	0.021
rs266729	0.029	0.047	0.03	-	1.0	0.418	0.135	0.194	0.869	0.884	0.241	0.059	0.641	0.763	0.625	0.634	0.713	0.517
rs182052	0.044	0.072	0.047	0.646	-	0.903	0.052	0.075	0.858	0.867	0.33	0.006	0.519	0.419	0.48	0.372	0.435	0.426
rs822393	0.026	0.042	0.027	0.159	0.48	-	0.98	1.0	0.981	1.0	0.287	0.019	0.411	0.227	0.343	0.276	0.259	0.405
rs822395	0.138	0.224	0.145	0.014	0.001	0.17	-	0.995	1.0	0.851	0.251	0.583	0.445	0.186	0.439	0.077	0.25	0.374
rs822396	0.017	0.525	0.018	0.022	0.002	0.077	0.431	-	1.0	0.784	0.929	0.869	0.867	0.734	0.876	0.791	0.778	0.704
rs7627128	0.015	0.025	0.016	0.406	0.255	0.568	0.105	0.046	-	1.0	0.221	0.18	0.605	0.749	0.566	0.693	0.677	0.595
rs2036373	0.002	0.002	0.005	0.017	0.026	0.02	0.022	0.008	0.012	-	1.0	1.0	0.768	0.926	0.782	0.939	0.924	0.78
rs17366568	0.001	0.017	0.011	0.021	0.025	0.033	0.017	0.027	0.033	0.008	-	0.981	1.0	0.683	1.0	0.199	0.69	1.0
rs17846866	0.006	0.009	0.006	0.0	0.0	0.0	0.05	0.012	0.0	0.004	0.517	-	0.863	0.496	0.795	0.953	0.485	0.817
rs2241766	0.001	0.015	0.0	0.019	0.019	0.007	0.012	0.02	0.009	0.004	0.017	0.007	-	1.0	0.988	0.889	0.917	0.954
rs1501299	0.066	0.047	0.118	0.094	0.044	0.008	0.007	0.051	0.049	0.118	0.027	0.008	0.051	-	1.0	0.992	0.997	0.949
rs2241767	0.001	0.014	0.001	0.017	0.016	0.005	0.011	0.02	0.008	0.004	0.016	0.005	0.938	0.049	-	0.985	1.0	0.976
rs3821799	0.044	0.093	0.083	0.128	0.068	0.022	0.003	0.116	0.082	0.062	0.007	0.081	0.114	0.5	0.135	-	0.992	0.891
rs3774261	0.066	0.077	0.109	0.13	0.075	0.016	0.021	0.09	0.063	0.074	0.044	0.012	0.151	0.628	0.173	0.791	-	0.896
rs1063539	0.0	0.015	0.0	0.013	0.013	0.007	0.009	0.014	0.009	0.004	0.017	0.006	0.885	0.047	0.89	0.118	0.148	-

Pairwise linkage disequilibrium D'-values are shown above the diagonal in the upper right triangle, r²-values are shown below the diagonal in the lower left triangle. *ADIPOQ*: adiponectin gene; SNP: single nucleotide polymorphism.
