# Supplementary material for: Adiponectin Gene Polymorphisms: A Case–Control Study on Their Role in Late-Onset Alzheimer’s Disease Risk
Source: Life (Basel). 2024 Mar 7;14(3):346. doi: 10.3390/life14030346 (PMC10971943; doi:10.3390/life14030346)
Supplement: Supplementary file 1 [file life-14-00346-s001.zip › Supplementary Table S4.pdf]

Supplementary Table S4. Hardy-Weinberg equilibrium values in patient and control populations.

ADIPOQ SNPs	**Controls** **HWE p / χ^2 Values**	**LOAD Patients** **HWE p / χ^2 Values**
rs822387	0.12 / 2.45	0.31 / 1.01
rs860291	0.20 / 1.66	0.60 / 0.27
rs17300539	0.086 / 3.01	0.57 / 0.32
rs266729	0.73 / 0.12	0.43 / 0.63
rs182052	0.66 / 0.19	0.48 / 0.49
rs822393	0.66 / 0.20	0.14 / 2.22
rs822395	0.41 / 0.67	0.48 / 0.49
rs822396	0.74 / 0.11	0.81 / 0.06
rs7627128	0.86 / 0.03	0.91 / 0.01
rs2036373	0.60 / 0.27	0.26 / 1.28
rs17366568	0.14 / 2.16	0.64 / 0.22
rs17846866	0.80 / 0.06	0.12 / 2.37
rs2241766	0.96 / 0.003	0.72 / 0.13
rs1501299	0.58 / 0.30	0.65 / 0.20
rs2241767	0.84 / 0.04	0.22 / 1.50
rs3821799	0.26 / 1.25	0.97 / 0.001
rs3774261	0.26 / 1.29	0.97 / 0.001
rs1063539	0.89 / 0.02	0.35 / 0.86

ADIPOQ: adiponectin gene; HWE: Hardy-Weinberg equilibrium; LOAD: late-onset Alzheimer's disease; SNP: single nucleotide polymorphism.
