# Supplementary material for: Adiponectin Gene Polymorphisms: A Case–Control Study on Their Role in Late-Onset Alzheimer’s Disease Risk
Source: Life (Basel). 2024 Mar 7;14(3):346. doi: 10.3390/life14030346 (PMC10971943; doi:10.3390/life14030346)
Supplement: Supplementary file 1 [file life-14-00346-s001.zip › Supplementary Table S5.pdf]

Supplementary Table S5. Association between *ADIPOQ* SNPs and LOAD risk in the whole study population.

Allele / Genotype	LOAD (n = 385)	Controls (n = 533)	Genetic Model	Crude Analysis p-Value	Crude Analysis OR (95% CI)	Logistic Regression Analysis[a] p-Value	Logistic Regression Analysis[a] OR (95% CI)
**rs822387**			Allele contrast (C vs. T)	0.81	0.96 (0.66–1.38)	0.81	0.96 (0.66–1.38)
T	718 (93.25%)	991 (92.96%)	Codominant (TC vs. TT)	0.96	0.98 (0.65–1.46)	0.83	0.95 (0.62–1.47)
C	52 (6.75%)	75 (7.04%)	Codominant (CC vs. TT)		0.83 (0.20–3.48)		0.64 (0.13–3.02)
TT	336 (87.27%)	463 (86.87%)	Dominant (TC + CC vs. TT)	0.86	0.96 (0.65–1.43)	0.72	0.93 (0.61–1.41)
TC	46 (11.95%)	65 (12.20%)	Recessive (CC vs. TT + TC)	0.80	0.83 (0.20–3.49)	0.57	0.64 (0.14–3.03)
CC	3 (0.78%)	5 (0.94%)	Over-dominant (TC vs. TT + CC)	0.91	0.98 (0.65–1.46)	0.84	0.96 (0.62–1.47)
			Log-additive	0.82	0.96 (0.67–1.37)	0.64	0.91 (0.62–1.34)
**rs860291**			Allele contrast (T vs. C)	0.49	1.11 (0.82–1.49)	0.49	1.11 (0.82–1.49)
C	682 (88.57%)	955 (89.59%)	Codominant (CT vs. CC)	0.65	1.08 (0.78–1.49)	0.73	1.07 (0.76–1.52)
T	88 (11.43%)	111 (10.41%)	Codominant (TT vs. CC)		1.88 (0.42–8.47)		1.78 (0.35–9.10)
CC	301 (78.18%)	425 (79.74%)	Dominant (CT + TT vs. CC)	0.57	1.10 (0.80–1.51)	0.62	1.09 (0.77–1.54)
CT	80 (20.78%)	105 (19.70%)	Recessive (TT vs. CC + CT)	0.42	1.85 (0.41–8.34)	0.50	1.76 (0.34–8.94)
TT	4 (1.04%)	3 (0.56%)	Over-dominant (CT vs. CC + TT)	0.69	1.07 (0.77–1.48)	0.72	1.07 (0.75–1.51)
			Log-additive	0.48	1.12 (0.82–1.51)	0.54	1.11 (0.80–1.53)
**rs17300539**			Allele contrast (A vs. G)	0.38	0.85 (0.59–1.22)	0.38	0.85 (0.59–1.22)
G	719 (93.38%)	984 (92.31%)	Codominant (GA vs. GG)	0.27	0.95 (0.65–1.41)	0.18	0.94 (0.62–1.44)
A	51 (6.62%)	82 (7.69%)	Codominant (AA vs. GG)		0.23 (0.03–1.90)		0.17 (0.02–1.53)
GG	335 (87.01%)	457 (85.74%)	Dominant (GA + AA vs. GG)	0.58	0.90 (0.61–1.32)	0.52	0.87 (0.58–1.32)
GA	49 (12.73%)	70 (13.13%)	Recessive (AA vs. GG + GA)	0.11	0.23 (0.03–1.91)	0.069	0.17 (0.02–1.55)
AA	1 (0.26%)	6 (1.13%)	Over-dominant (GA vs. GG + AA)	0.86	0.96 (0.65–1.43)	0.83	0.95 (0.62–1.46)
			Log-additive	0.39	0.86 (0.60–1.22)	0.32	0.82 (0.56–1.21)
**rs266729**			Allele contrast (G vs. C)	0.83	0.98 (0.79–1.20)	0.83	0.98 (0.79–1.20)
C	559 (72.60%)	769 (72.14%)	Codominant (CG vs. CC)	0.91	0.94 (0.72–1.24)	0.71	0.89 (0.66–1.19)
G	211 (27.40%)	297 (27.86%)	Codominant (GG vs. CC)		1.01 (0.62–1.65)		1.00 (0.59–1.69)
CC	206 (53.51%)	279 (52.34%)	Dominant (CG + GG vs. CC)	0.73	0.95 (0.73–1.24)	0.49	0.91 (0.68–1.20)
CG	147 (38.18%)	211 (39.59%)	Recessive (GG vs. CC + CG)	0.89	1.03 (0.64–1.67)	0.84	1.05 (0.64–1.75)
GG	32 (8.31%)	43 (8.07%)	Over-dominant (CG vs. CC + GG)	0.67	0.94 (0.72–1.23)	0.41	0.89 (0.66–1.18)
			Log-additive	0.83	0.98 (0.80–1.20)	0.66	0.95 (0.76–1.18)
**rs182052**			Allele contrast (A vs. G)	0.52	1.06 (0.88–1.29)	0.52	1.06 (0.88–1.29)
G	477 (61.95%)	676 (63.41%)	Codominant (GA vs. GG)	0.58	0.97 (0.73–1.30)	0.63	0.94 (0.69–1.27)
A	293 (38.05%)	390 (36.59%)	Codominant (AA vs. GG)		1.20 (0.80–1.80)		1.15 (0.75–1.77)
GG	151 (39.22%)	212 (39.77%)	Dominant (GA + AA vs. GG)	0.87	1.02 (0.78–1.34)	0.91	0.98 (0.74–1.31)
GA	175 (45.46%)	252 (47.28%)	Recessive (AA vs. GG + GA)	0.31	1.22 (0.84–1.77)	0.39	1.19 (0.80–1.78)
AA	59 (15.32%)	69 (12.95%)	Over-dominant (GA vs. GG + AA)	0.58	0.93 (0.71–1.21)	0.48	0.90 (0.68–1.20)

			Log-additive	0.52	1.06 (0.88–1.29)	0.72	1.04 (0.85–1.27)
**rs822393**			Allele contrast (T vs. C)	0.50	1.07 (0.87–1.33)	0.50	1.07 (0.87–1.33)
C	565 (73.38%)	797 (74.77%)	Codominant (CT vs. CC)	0.30	0.94 (0.71–1.24)	0.35	0.99 (0.74–1.34)
T	205 (26.62%)	269 (25.23%)	Codominant (TT vs. CC)		1.43 (0.85–2.40)		1.48 (0.85–2.57)
CC	213 (55.33%)	296 (55.53%)	Dominant (CT + TT vs. CC)	0.95	1.01 (0.77–1.31)	0.68	1.06 (0.80–1.41)
CT	139 (36.10%)	205 (38.46%)	Recessive (TT vs. CC + CT)	0.14	1.47 (0.89–2.43)	0.15	1.49 (0.87–2.55)
TT	33 (8.57%)	32 (6.00%)	Over-dominant (CT vs. CC + TT)	0.47	0.90 (0.69–1.19)	0.72	0.95 (0.71–1.27)
			Log-additive	0.51	1.07 (0.87–1.32)	0.36	1.11 (0.89–1.39)
**rs822395**			Allele contrast (C vs. A)	0.77	1.03 (0.85–1.25)	0.77	1.03 (0.85–1.25)
A	507 (65.84%)	709 (66.51%)	Codominant (AC vs. AA)	0.96	1.03 (0.78–1.36)	0.97	1.00 (0.74–1.35)
C	263 (34.16%)	357 (33.49%)	Codominant (CC vs. AA)		1.06 (0.69–1.62)		0.95 (0.60–1.50)
AA	170 (44.15%)	240 (45.03%)	Dominant (AC + CC vs. AA)	0.79	1.04 (0.80–1.35)	0.93	0.99 (0.74–1.31)
AC	167 (43.38%)	229 (42.96%)	Recessive (CC vs. AA + AC)	0.83	1.04 (0.70–1.56)	0.81	0.95 (0.62–1.46)
CC	48 (12.47%)	64 (12.01%)	Over-dominant (AC vs. AA + CC)	0.90	1.02 (0.78–1.33)	0.95	1.01 (0.76–1.34)
			Log-additive	0.77	1.03 (0.85–1.25)	0.86	0.98 (0.80–1.21)
**rs822396**			Allele contrast (G vs. A)	0.72	1.04 (0.82–1.33)	0.72	1.04 (0.82–1.33)
A	627 (81.43%)	875 (82.08%)	Codominant (AG vs. AA)	0.87	1.01 (0.76–1.35)	0.86	0.93 (0.68–1.27)
G	143 (18.57%)	191 (17.92%)	Codominant (GG vs. AA)		1.22 (0.59–2.55)		1.09 (0.49–2.42)
AA	256 (66.49%)	358 (67.17%)	Dominant (AG + GG vs. AA)	0.83	1.03 (0.78–1.36)	0.70	0.94 (0.70–1.27)
AG	115 (29.87%)	159 (29.83%)	Recessive (GG vs. AA + AG)	0.60	1.22 (0.59–2.53)	0.79	1.11 (0.50–2.46)
GG	14 (3.64%)	16 (3.00%)	Over-dominant (AG vs. AA + GG)	0.99	1.00 (0.75–1.33)	0.61	0.92 (0.68–1.26)
			Log-additive	0.72	1.04 (0.82–1.33)	0.80	0.97 (0.75–1.25)
**rs7627128**			Allele contrast (A vs. C)	0.51	0.92 (0.72–1.18)	0.51	0.92 (0.72–1.18)
C	644 (83.64%)	879 (82.46%)	Codominant (CA vs. CC)	0.79	0.93 (0.70–1.25)	0.83	0.95 (0.70–1.31)
A	126 (16.36%)	187 (17.54%)	Codominant (AA vs. CC)		0.79 (0.36–1.76)		0.79 (0.34–1.83)
CC	269 (69.87%)	363 (68.10%)	Dominant (CA + AA vs. CC)	0.57	0.92 (0.69–1.22)	0.68	0.94 (0.69–1.27)
CA	106 (27.53%)	153 (28.71%)	Recessive (AA vs. CC + CA)	0.60	0.81 (0.37–1.79)	0.60	0.80 (0.35–1.85)
AA	10 (2.60%)	17 (3.19%)	Over-dominant (CA vs. CC + AA)	0.70	0.94 (0.70–1.26)	0.82	0.96 (0.70–1.32)
			Log-additive	0.51	0.92 (0.72–1.18)	0.59	0.93 (0.71–1.21)
**rs2036373**			Allele contrast (G vs. T)	0.94	0.98 (0.65–1.48)	0.94	0.98 (0.65–1.48)
T	728 (94.55%)	1,007 (94.47%)	Codominant (TG vs. TT)	0.58	1.02 (0.67–1.56)	0.49	1.17 (0.74–1.84)
G	42 (5.45%)	59 (5.53%)	Codominant (GG vs. TT)		0.46 (0.02–11.37)		NA
TT	343 (89.09%)	475 (89.12%)	Dominant (TG + GG vs. TT)	0.99	1.00 (0.66–1.53)	0.56	1.15 (0.73–1.80)
TG	42 (10.91%)	57 (10.69%)	Recessive (GG vs. TT + TG)	0.30	0.46 (0.02–11.34)	0.33	NA
GG	0 (0.00%)	1 (0.19%)	Over-dominant (TG vs. TT + GG)	0.92	1.02 (0.67–1.56)	0.50	1.17 (0.74–1.85)
			Log-additive	0.94	0.98 (0.65–1.49)	0.62	1.12 (0.72–1.75)
**rs17366568**			Allele contrast (A vs. G)	0.32	0.86 (0.65–1.15)	0.32	0.86 (0.65–1.15)
G	683 (88.70%)	929 (87.15%)	Codominant (GA vs. GG)	0.49	0.83 (0.60–1.14)	0.72	0.87 (0.62–1.22)

A	87 (11.30%)	137 (12.85%)	Codominant (AA vs. GG)		1.06 (0.28–3.99)		1.02 (0.24–4.26)
GG	302 (78.44%)	401 (75.23%)	Dominant (GA + AA vs. GG)	0.26	0.83 (0.61–1.14)	0.43	0.87 (0.63–1.22)
GA	79 (20.52%)	127 (23.83%)	Recessive (AA vs. GG + GA)	0.88	1.11 (0.30–4.16)	0.95	1.05 (0.25–4.39)
AA	4 (1.04%)	5 (0.94%)	Over-dominant (GA vs. GG + AA)	0.23	0.83 (0.60–1.13)	0.42	0.87 (0.62–1.22)
			Log-additive	0.30	0.86 (0.64–1.15)	0.47	0.89 (0.65–1.22)
**rs17846866**			Allele contrast (G vs. T)	0.61	1.10 (0.76–1.58)	0.61	1.10 (0.76–1.58)
T	714 (92.73%)	995 (93.34%)	Codominant (TG vs. TT)	0.24	1.18 (0.80–1.73)	0.25	1.23 (0.82–1.87)
G	56 (7.27%)	71 (6.66%)	Codominant (GG vs. TT)		0.28 (0.01–5.90)		NA
TT	329 (85.45%)	464 (87.05%)	Dominant (TG + GG vs. TT)	0.49	1.14 (0.78–1.67)	0.39	1.20 (0.80–1.81)
TG	56 (14.55%)	67 (12.57%)	Recessive (GG vs. TT + TG)	0.14	0.27 (0.01–5.76)	0.18	NA
GG	0 (0.00%)	2 (0.38%)	Over-dominant (TG vs. TT + GG)	0.39	1.18 (0.81–1.73)	0.31	1.24 (0.82–1.87)
			Log-additive	0.60	1.10 (0.76–1.60)	0.48	1.15 (0.77–1.72)
**rs2241766**			Allele contrast (G vs. T)	0.72	1.06 (0.78–1.43)	0.72	1.06 (0.78–1.43)
T	685 (88.96%)	954 (89.49%)	Codominant (TG vs. TT)	0.89	1.08 (0.78–1.51)	0.88	1.10 (0.77–1.56)
G	85 (11.04%)	112 (10.51%)	Codominant (GG vs. TT)		0.94 (0.26–3.35)		1.00 (0.25–3.92)
TT	304 (78.96%)	427 (80.11%)	Dominant (TG + GG vs. TT)	0.67	1.07 (0.78–1.48)	0.63	1.09 (0.77–1.54)
TG	77 (20.00%)	100 (18.76%)	Recessive (GG vs. TT + TG)	0.90	0.92 (0.26–3.29)	0.98	0.98 (0.25–3.85)
GG	4 (1.04%)	6 (1.13%)	Over-dominant (TG vs. TT + GG)	0.64	1.08 (0.78–1.51)	0.61	1.10 (0.77–1.56)
			Log-additive	0.72	1.06 (0.78–1.43)	0.66	1.08 (0.78–1.48)
**rs1501299**			Allele contrast (T vs. G)	0.53	1.07 (0.87–1.31)	0.53	1.07 (0.87–1.31)
G	535 (69.48%)	755 (70.83%)	Codominant (GT vs. GG)	0.65	1.14 (0.87–1.50)	0.21	1.30 (0.97–1.76)
T	235 (30.52%)	311 (29.17%)	Codominant (TT vs. GG)		1.04 (0.64–1.68)		1.08 (0.65–1.81)
GG	184 (47.79%)	270 (50.66%)	Dominant (GT + TT vs. GG)	0.39	1.12 (0.86–1.46)	0.11	1.26 (0.95–1.68)
GT	167 (43.38%)	215 (40.34%)	Recessive (TT vs. GG + GT)	0.93	0.98 (0.62–1.55)	0.87	0.96 (0.59–1.57)
TT	34 (8.83%)	48 (9.00%)	Over-dominant (GT vs. GG + TT)	0.36	1.13 (0.87–1.48)	0.084	1.29 (0.97–1.72)
			Log-additive	0.54	1.07 (0.87–1.30)	0.24	1.14 (0.92–1.41)
**rs2241767**			Allele contrast (G vs. A)	0.84	1.03 (0.76–1.40)	0.84	1.03 (0.76–1.40)
A	689 (89.48%)	957 (89.77%)	Codominant (AG vs. AA)	0.49	1.12 (0.80–1.56)	0.63	1.12 (0.78–1.60)
G	81 (10.52%)	109 (10.22%)	Codominant (GG vs. AA)		0.47 (0.09–2.34)		0.55 (0.10–2.99)
AA	306 (79.48%)	430 (80.67%)	Dominant (AG + GG vs. AA)	0.65	1.08 (0.78–1.50)	0.65	1.09 (0.76–1.54)
AG	77 (20.00%)	97 (18.20%)	Recessive (GG vs. AA + AG)	0.31	0.46 (0.09–2.28)	0.45	0.54 (0.10–2.93)
GG	2 (0.52%)	6 (1.13%)	Over-dominant (AG vs. AA + GG)	0.49	1.12 (0.81–1.57)	0.53	1.12 (0.79–1.61)
			Log-additive	0.84	1.03 (0.76–1.40)	0.79	1.05 (0.75–1.46)
**rs3821799**			Allele contrast (T vs. C)	0.62	1.05 (0.87–1.26)	0.62	1.05 (0.87–1.26)
C	415 (53.90%)	587 (55.07%)	Codominant (CT vs. CC)	0.69	1.14 (0.84–1.55)	0.29	1.30 (0.93–1.80)
T	355 (46.10%)	479 (44.93%)	Codominant (TT vs. CC)		1.08 (0.74–1.56)		1.21 (0.81–1.81)
CC	112 (29.09%)	168 (31.52%)	Dominant (CT + TT vs. CC)	0.43	1.12 (0.84–1.49)	0.13	1.27 (0.93–1.73)
CT	191 (49.61%)	251 (47.09%)	Recessive (TT vs. CC + CT)	0.97	0.99 (0.72–1.37)	0.85	1.03 (0.73–1.46)

TT	82 (21.30%)	114 (21.39%)	Over-dominant (CT vs. CC + TT)	0.45	1.11 (0.85–1.44)	0.21	1.20 (0.90–1.59)
			Log-additive	0.62	1.05 (0.87–1.26)	0.28	1.12 (0.92–1.36)
**rs3774261**			Allele contrast (A vs. G)	0.48	1.07 (0.89–1.29)	0.48	1.07 (0.89–1.29)
G	454 (58.96%)	646 (60.60%)	Codominant (GA vs. GG)	0.61	1.16 (0.87–1.55)	0.24	1.30 (0.95–1.78)
A	316 (41.04%)	420 (39.40%)	Codominant (AA vs. GG)		1.10 (0.75–1.62)		1.21 (0.80–1.84)
GG	134 (34.81%)	202 (37.90%)	Dominant (GA + AA vs. GG)	0.34	1.14 (0.87–1.50)	0.10	1.28 (0.95–1.72)
GA	186 (48.31%)	242 (45.40%)	Recessive (AA vs. GG + GA)	0.94	1.01 (0.71–1.44)	0.82	1.04 (0.72–1.52)
AA	65 (16.88%)	89 (16.70%)	Over-dominant (GA vs. GG + AA)	0.38	1.12 (0.86–1.46)	0.16	1.23 (0.92–1.63)
			Log-additive	0.49	1.07 (0.89–1.29)	0.21	1.14 (0.93–1.39)
**rs1063539**			Allele contrast (C vs. G)	0.85	1.03 (0.77–1.38)	0.85	1.03 (0.77–1.38)
G	684 (88.83%)	950 (89.12%)	Codominant (GC vs. GG)	0.79	1.08 (0.78–1.49)	0.93	1.06 (0.75–1.51)
C	86 (11.17%)	116 (10.88%)	Codominant (CC vs. GG)		0.70 (0.17–2.82)		0.86 (0.20–3.74)
GG	302 (78.44%)	423 (79.36%)	Dominant (GC + CC vs. GG)	0.74	1.06 (0.77–1.46)	0.79	1.05 (0.74–1.48)
GC	80 (20.78%)	104 (19.51%)	Recessive (CC vs. GG + GC)	0.59	0.69 (0.17–2.78)	0.83	0.85 (0.20–3.69)
CC	3 (0.78%)	6 (1.13%)	Over-dominant (GC vs. GG + CC)	0.64	1.08 (0.78–1.50)	0.74	1.06 (0.75–1.51)
			Log-additive	0.84	1.03 (0.76–1.39)	0.84	1.03 (0.75–1.43)

Allele and genotype counts are presented as absolute values with % in parentheses. [a]*P*, OR, and 95% CI values for genotype comparisons were adjusted for age, sex, *APOE* ε4 carrier status, hypertension, type 2 diabetes mellitus, and body mass index. *ADIPOQ*: adiponectin gene; *APOE*: apolipoprotein E gene; CI: confidence interval; LOAD: late-onset Alzheimer's disease; OR: odds ratio.
