# Supplementary material for: Adiponectin Gene Polymorphisms: A Case–Control Study on Their Role in Late-Onset Alzheimer’s Disease Risk
Source: Life (Basel). 2024 Mar 7;14(3):346. doi: 10.3390/life14030346 (PMC10971943; doi:10.3390/life14030346)
Supplement: Supplementary file 1 [file life-14-00346-s001.zip › Supplementary Table S6.pdf]

Supplementary Table S6. Association between *ADIPOQ* polymorphisms and LOAD risk in the *APOE* ε4-positive subpopulation.

ADIPOQ SNPs	***APOE* ε4+ LOAD** (n = 195)		***APOE* ε4+ Controls** (n = 101)		**Logistic Regression Analysis (Log-additive Model)**	
	**Genotypes**[a]	**MAF**	**Genotypes**[a]	**MAF**	***p*-Value**	**OR (95% CI)**
rs822387 T>C	167/25/3	0.0795	86/14/1	0.0792	0.92	1.03 (0.56–1.90)
rs860291 C>T	151/42/2	0.1179	84/16/1	0.0891	0.24	1.42 (0.79–2.55)
rs17300539 G>A	168/26/1	0.0718	84/15/2	0.0941	0.34	0.75 (0.41–1.36)
rs266729 C>G	98/83/14	0.2846	50/43/8	0.2921	0.90	0.98 (0.66–1.43)
rs182052 G>A	72/95/28	0.3872	38/49/14	0.3812	0.91	1.02 (0.72–1.46)
rs822393 C>T	112/67/16	0.2538	59/36/6	0.2376	0.72	1.07 (0.73–1.57)
rs822395 A>C	79/87/29	0.3718	47/40/14	0.3366	0.41	1.16 (0.82–1.64)
rs822396 A>G	123/64/8	0.2051	68/28/5	0.1881	0.60	1.12 (0.73–1.72)
rs7627128 C>A	135/56/4	0.1641	71/26/4	0.1683	0.96	0.99 (0.62–1.56)
rs2036373 T>G	177/18/0	0.0462	92/9/0	0.0446	0.98	1.01 (0.43–2.36)
rs17366568 G>A	151/42/2	0.1179	81/19/1	0.1040	0.59	1.17 (0.67–2.05)
rs17846866 T>G	167/28/0	0.0718	91/10/0	0.0495	0.33	1.46 (0.67–3.15)
rs2241766 T>G	155/38/2	0.1077	84/16/1	0.0891	0.47	1.24 (0.69–2.23)
rs1501299 G>T	100/80/15	0.2821	52/38/11	0.2970	0.76	0.94 (0.65–1.37)
rs2241767 A>G	156/38/1	0.1026	84/16/1	0.0891	0.62	1.16 (0.64–2.11)
rs3821799 C>T	62/96/37	0.4359	37/41/23	0.4307	0.93	1.02 (0.73–1.42)
rs3774261 G>A	75/90/30	0.3846	41/42/18	0.3861	0.99	1.00 (0.71–1.41)
rs1063539 G>C	155/39/1	0.1051	82/18/1	0.0990	0.89	1.04 (0.58–1.87)

[a]Genotype counts are presented as follows: major allele homozygotes/heterozygotes/minor allele homozygotes. Logistic regression analysis was adjusted for age, sex, hypertension, type 2 diabetes mellitus, and body mass index. *ADIPOQ*: adiponectin gene; *APOE*: apolipoprotein E gene; CI: confidence interval; LOAD: late-onset Alzheimer's disease; MAF: minor allele frequency; OR: odds ratio; SNP: single nucleotide polymorphism.
