# Supplementary material for: Adiponectin Gene Polymorphisms: A Case–Control Study on Their Role in Late-Onset Alzheimer’s Disease Risk
Source: Life (Basel). 2024 Mar 7;14(3):346. doi: 10.3390/life14030346 (PMC10971943; doi:10.3390/life14030346)
Supplement: Supplementary file 1 [file life-14-00346-s001.zip › Supplementary Table S7.pdf]

Supplementary Table S7. Association between *ADIPOQ* polymorphisms and LOAD risk in the *APOE* ε4-negative subpopulation.

ADIPOQ SNPs	*APOE* ε4- LOAD (n = 190)		*APOE* ε4- Controls (n = 432)		Logistic Regression Analysis (Log-additive Model)	
	Genotypes[a]	MAF	Genotypes[a]	MAF	*p*-Value	OR (95% CI)
rs822387 T>C	169/21/0	0.0553	377/51/4	0.0683	0.46	0.83 (0.50–1.38)
rs860291 C>T	150/38/2	0.1105	341/89/2	0.1076	0.90	0.97 (0.65–1.46)
rs17300539 G>A	167/23/0	0.0605	373/55/4	0.0729	0.60	0.88 (0.53–1.44)
rs266729 C>G	108/64/18	0.2632	229/168/35	0.2755	0.65	0.94 (0.72–1.23)
rs182052 G>A	79/80/31	0.3737	174/203/55	0.3623	0.71	1.05 (0.82–1.35)
rs822393 C>T	101/72/17	0.2789	237/169/26	0.2558	0.39	1.13 (0.86–1.49)
rs822395 A>C	91/80/19	0.3105	193/189/50	0.3345	0.37	0.89 (0.68–1.15)
rs822396 A>G	133/51/6	0.1658	290/131/11	0.1771	0.46	0.88 (0.63–1.23)
rs7627128 C>A	134/50/6	0.1632	292/127/13	0.1771	0.51	0.90 (0.65–1.24)
rs2036373 T>G	166/24/0	0.0632	383/48/1	0.0579	0.56	1.17 (0.70–1.97)
rs17366568 G>A	151/37/2	0.1079	320/108/4	0.1343	0.16	0.76 (0.51–1.12)
rs17846866 T>G	162/28/0	0.0737	373/57/2	0.0706	0.89	1.04 (0.64–1.68)
rs2241766 T>G	149/39/2	0.1132	343/84/5	0.1088	0.92	1.02 (0.69–1.51)
rs1501299 G>T	84/87/19	0.3289	218/177/37	0.2905	0.098	1.25 (0.96–1.63)
rs2241767 A>G	150/39/1	0.1079	346/81/5	0.1053	0.96	1.01 (0.68–1.51)
rs3821799 C>T	50/95/45	0.4868	131/210/91	0.4537	0.19	1.18 (0.92–1.50)
rs3774261 G>A	59/96/35	0.4368	161/200/71	0.3958	0.12	1.22 (0.95–1.56)
rs1063539 G>C	147/41/2	0.1184	341/86/5	0.1111	0.83	1.04 (0.71–1.54)

[a]Genotype counts are presented as follows: major allele homozygotes/heterozygotes/minor allele homozygotes. Logistic regression analysis was adjusted for age, sex, hypertension, type 2 diabetes mellitus, and body mass index. *ADIPOQ*: adiponectin gene; *APOE*: apolipoprotein E gene; CI: confidence interval; LOAD: late-onset Alzheimer's disease; MAF: minor allele frequency; OR: odds ratio; SNP: single nucleotide polymorphism.
