# Supplementary material for: Adiponectin Gene Polymorphisms: A Case–Control Study on Their Role in Late-Onset Alzheimer’s Disease Risk
Source: Life (Basel). 2024 Mar 7;14(3):346. doi: 10.3390/life14030346 (PMC10971943; doi:10.3390/life14030346)
Supplement: Supplementary file 1 [file life-14-00346-s001.zip › Supplementary Table S8.pdf]

Supplementary Table S8. Association between *ADIPOQ* polymorphisms and LOAD risk in female subjects.

ADIPOQ SNPs	Females with LOAD (n = 243)		Female Controls (n = 320)		Logistic Regression Analysis (Log-additive Model)	
	Genotypes[a]	MAF	Genotypes[a]	MAF	*p*-Value	OR (95% CI)
rs822387 T>C	213/27/3	0.0679	279/38/3	0.0688	0.80	0.94 (0.58–1.53)
rs860291 C>T	185/54/4	0.1276	244/74/2	0.1219	0.81	1.05 (0.71–1.55)
rs17300539 G>A	213/29/1	0.0638	277/40/3	0.0719	0.42	0.81 (0.49–1.34)
rs266729 C>G	132/90/21	0.2716	167/125/28	0.2828	0.56	0.92 (0.70–1.22)
rs182052 G>A	97/110/36	0.3745	127/150/43	0.3688	0.92	0.99 (0.76–1.28)
rs822393 C>T	134/90/19	0.2634	180/122/18	0.2469	0.57	1.09 (0.81–1.45)
rs822395 A>C	106/101/36	0.3560	138/141/41	0.3484	0.79	0.97 (0.74–1.25)
rs822396 A>G	156/77/10	0.1996	200/108/12	0.2063	0.52	0.90 (0.65–1.24)
rs7627128 C>A	170/67/6	0.1626	221/88/11	0.1719	0.57	0.91 (0.65–1.27)
rs2036373 T>G	217/26/0	0.0535	283/36/1	0.0594	0.95	0.98 (0.56–1.72)
rs17366568 G>A	195/46/2	0.1029	250/67/3	0.1141	0.31	0.81 (0.53–1.22)
rs17846866 T>G	212/31/0	0.0638	285/34/1	0.0563	0.77	1.09 (0.63–1.87)
rs2241766 T>G	193/46/4	0.1111	256/59/5	0.1078	0.57	1.12 (0.75–1.67)
rs1501299 G>T	126/95/22	0.2860	165/129/26	0.2828	0.54	1.09 (0.83–1.45)
rs2241767 A>G	193/48/2	0.1070	257/58/5	0.1063	0.67	1.09 (0.73–1.65)
rs3821799 C>T	80/111/52	0.4424	106/150/64	0.4344	0.40	1.11 (0.87–1.43)
rs3774261 G>A	91/112/40	0.3951	124/143/53	0.3891	0.38	1.12 (0.87–1.45)
rs1063539 G>C	190/50/3	0.1152	252/64/4	0.1125	0.64	1.10 (0.74–1.64)

[a]Genotype counts are presented as follows: major allele homozygotes/heterozygotes/minor allele homozygotes. Logistic regression analysis was adjusted for age, *APOE* ε4 carrier status, hypertension, type 2 diabetes mellitus, and body mass index. *ADIPOQ*: adiponectin gene; *APOE*: apolipoprotein E gene; CI: confidence interval; LOAD: late-onset Alzheimer's disease; MAF: minor allele frequency; OR: odds ratio; SNP: single nucleotide polymorphism.
