# Supplementary material for: Adiponectin Gene Polymorphisms: A Case–Control Study on Their Role in Late-Onset Alzheimer’s Disease Risk
Source: Life (Basel). 2024 Mar 7;14(3):346. doi: 10.3390/life14030346 (PMC10971943; doi:10.3390/life14030346)
Supplement: Supplementary file 1 [file life-14-00346-s001.zip › Supplementary Table S9.pdf]

Supplementary Table S9. Association between *ADIPOQ* polymorphisms and LOAD risk in male subjects.

ADIPOQ SNPs	Males with LOAD (n = 142)		Male Controls (n = 213)		Logistic Regression Analysis (Log-additive Model)	
	Genotypes[a]	MAF	Genotypes[a]	MAF	*p*-Value	OR (95% CI)
rs822387 T>C	123/19/0	0.0669	184/27/2	0.0728	0.66	0.87 (0.46–1.62)
rs860291 C>T	116/26/0	0.0915	181/31/1	0.0775	0.46	1.25 (0.70–2.24)
rs17300539 G>A	122/20/0	0.0704	180/30/3	0.0845	0.53	0.83 (0.45–1.50)
rs266729 C>G	74/57/11	0.2782	112/86/15	0.2723	0.97	1.01 (0.70–1.44)
rs182052 G>A	54/65/23	0.3908	85/102/26	0.3615	0.47	1.13 (0.81–1.57)
rs822393 C>T	79/49/14	0.2711	116/83/14	0.2606	0.46	1.14 (0.80–1.63)
rs822395 A>C	64/66/12	0.3169	102/88/23	0.3146	0.99	1.00 (0.71–1.41)
rs822396 A>G	100/38/4	0.1620	158/51/4	0.1385	0.63	1.12 (0.71–1.74)
rs7627128 C>A	99/39/4	0.1655	142/65/6	0.1808	0.87	0.96 (0.63–1.48)
rs2036373 T>G	126/16/0	0.0563	192/21/0	0.0493	0.36	1.41 (0.68–2.94)
rs17366568 G>A	107/33/2	0.1303	151/60/2	0.1502	0.96	0.99 (0.61–1.61)
rs17846866 T>G	117/25/0	0.0880	179/33/1	0.0822	0.53	1.21 (0.67–2.19)
rs2241766 T>G	111/31/0	0.1092	171/41/1	0.1009	0.97	1.01 (0.58–1.74)
rs1501299 G>T	58/72/12	0.3380	105/86/22	0.3052	0.27	1.21 (0.86–1.71)
rs2241767 A>G	113/29/0	0.1021	173/39/1	0.0962	0.95	0.98 (0.56–1.72)
rs3821799 C>T	32/80/30	0.4930	62/101/50	0.4718	0.49	1.12 (0.81–1.55)
rs3774261 G>A	43/74/25	0.4366	78/99/36	0.4014	0.35	1.17 (0.84–1.61)
rs1063539 G>C	112/30/0	0.1056	171/40/2	0.1033	0.82	0.94 (0.54–1.62)

[a]Genotype counts are presented as follows: major allele homozygotes/heterozygotes/minor allele homozygotes. Logistic regression analysis was adjusted for age, *APOE* ε4 carrier status, hypertension, type 2 diabetes mellitus, and body mass index. *ADIPOQ*: adiponectin gene; *APOE*: apolipoprotein E gene; CI: confidence interval; LOAD: late-onset Alzheimer's disease; MAF: minor allele frequency; OR: odds ratio; SNP: single nucleotide polymorphism.
